# A multiscale, Bayesian inference approach to augment mechanistic models of cell signaling with machine-learning predictions of binding affinity

**DOI:** 10.1371/journal.pcbi.1014321

**Published:** 2026-06-05

**Authors:** Holly A. Huber, Stacey D. Finley

**Affiliations:** 1 Alfred E. Mann Department of Biomedical Engineering, University of Southern California, Los Angeles, California, United States of America; 2 Department of Quantitative and Computational Biology, University of Southern California, Los Angeles, California, United States of America; 3 Mork Family Department of Chemical Engineering and Materials Science, University of Southern California, Los Angeles, California, United States of America; University of Birmingham, UNITED KINGDOM OF GREAT BRITAIN AND NORTHERN IRELAND

## Abstract

Computational models in systems biology are often underdetermined—that is, there is little data relative to the complexity and size of the model. This lack of data is primarily due to limits in our ability to observe specific biological systems and restricts the utility of computational models. To reduce this uncertainty, recent methods have explored augmenting parameter inference of systems biology models with predictions from machine learning models. Such approaches expand the pool of data that is applicable for the inference problem. Here, we explore augmenting the parameter inference of intracellular signaling models. We choose to investigate signaling because experimental measurements of the variables of interest, protein dynamics, are still quite limited. To investigate, we propose a novel, multiscale, Bayesian inference approach that augments traditional signaling data with predictions of binding affinity. These predictions are generated using a machine learning pipeline with measurements of amino acid sequence, from the Universal Protein Resource, or protein structure, from the Protein Data Bank, as inputs. We find that we can successfully integrate these measurements into the inference problem using our novel framework. Excitingly, this integration significantly improves the parameter estimates of signaling models. We demonstrate that how much this improvement impacts predictions of signaling depends on the sensitivity of the prediction to perturbations in the parameter values. Overall, the framework we establish here improves the parameter inference of intracellular signaling models by successfully bridging data on protein sequence and structure with systems-level signaling.

## Author summary

Computational models of cell signaling have provided mechanistic insights into complex biological systems, including in physiological and disease settings. Accurate and predictive modeling critically depends on the precise estimation of model parameters, which is often hindered by the limited availability of experimental data. In this study, we present a novel multiscale probabilistic inference framework that broadens the scope of data types that can be leveraged for parameter estimation for models of cell signaling. The framework integrates a machine learning pipeline with a generalizable parameter inference approach, enabling the use of experimental data across scales. Specifically, we demonstrate that incorporating protein amino acid sequence and 3D structural data enhances parameter estimation compared to traditional measurements such as protein concentrations over time. Improving parameter estimation increases the robustness and applicability of cell signaling models. Ultimately, our framework enables more biologically grounded simulations by bridging data on sequence and structure with systems-level signaling.

## 1. Introduction

The application of computational modeling to biological systems has generated a substantial surge in discoveries and insights into these systems; however, especially as systems become more complex, accurately determining computational models remains challenging [1]. For example, it can be challenging to parameterize systems biology models, as we often do not have direct measurements of model parameters. Instead, parameters must be inferred using noisy, partial observations of the system of interest. For instance, a system of ordinary differential equations (ODEs) may be used to model intracellular signaling [2]. Such mechanistic models are useful, having been applied to optimize CAR-T cell therapies or elucidate control mechanisms of cellular responses [3,4]. However, we rarely have observations of all the model parameters—such as binding affinities or protein shuttling rates. Instead, these parameters must be inferred using observations of the model variables: protein concentrations over time. Timeseries measurements of protein concentrations are often sparse, limited to relative concentrations, and noisy. When such measurements are used for parameter estimation, these limitations can result in significant uncertainty in the model predictions, which limits the applicability of the model [5].

To reduce this uncertainty, recent approaches have explored augmenting noisy or partial observations of a biological system of interest with predictions from machine learning (ML) models. For example, ML models have been leveraged to predict the parameters of models of cellular metabolism and steady-state signaling using amino acid sequence or protein structure [6–8]. We note that the latter study on steady state signaling was tested on only one protein in the system, limiting the generalizability of the results.

Traditionally, data on protein sequence and structure has not been available for the parameter estimation of such systems biology models, due to the difference in scale between the variables of systems biology models and protein sequence or structure. ML models change this availability, potentially enabling the use of rich databases such as the Protein Data Bank (PDB), which contains data on protein structure, and Universal Protein Resource (UniProt), which contains data on amino acid sequence, for new systems biology modeling applications.

Here, we hypothesize that despite differences in scale, measurements of protein structure and amino acid sequence can also facilitate the parameter inference of ODE models of cell signaling. To test this hypothesis, we propose a novel, multiscale, Bayesian modeling framework that expands the experimental measurements that may be integrated into the parameter estimation of cell signaling models. Excitingly, ours is the first such approach tested on dynamic signaling models. Furthermore, we aim to increase the generalizability of our results by presenting an expanded sample size.

**Fig 1:**
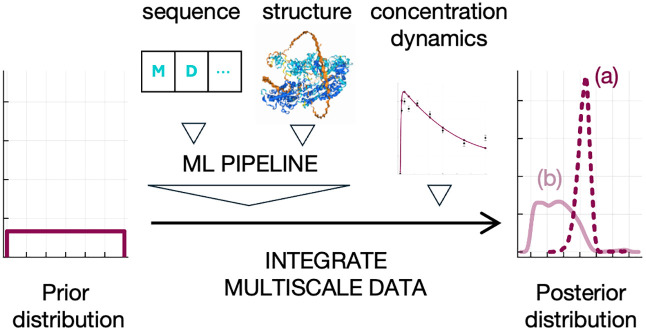
Multiscale Bayesian modeling framework. Starting with the same range of possible parameter values—prior distribution—for the ODE model of cell signaling, we integrate data from different scales into our Bayesian parameter inference using a ML pipeline. We perform inference using two data sets. One dataset comprises only concentration dynamics. The other comprises concentration dynamics as well as amino acid sequence and protein structure. We may then compare the difference between the plausible parameter values—posterior distribution—with, **(a)**, and without, **(b)**, measurements of sequence and structure.

Fig 1 illustrates our proposed framework. The framework comprises a ML pipeline that predicts the binding affinity parameters of signaling models using protein structure or amino acid sequence. This ML prediction is incorporated into the likelihood function of a Bayesian parameter inference routine. Using Bayesian inference allows us to not only estimate model parameters, but also quantify the information gained from augmenting parameter inference with sequence and structure [9].

Indeed, Bayesian inference characterizes a distribution of plausible parameter values—the posterior distribution—given a particular dataset. To quantify the information gained from a particular dataset, one can compare the distribution of plausible parameter values before and after integrating data using statistical distance metrics. One common distance metric used to quantify the difference between distributions is Kullback Leibler (KL) divergence [10]. The more the posterior distribution changes, the larger the KL divergence becomes, indicating that more information is gained from the data. KL divergence has been applied in experimental design and hypothesis generation [11–13]. Here, we use it to quantify the information gained from incorporating experimental observations of sequence or structure into the parameter inference of cell signaling models.

We test our approach on two, well characterized ODE models of intracellular signaling: Epidermal Growth Factor Receptor (EGFR) and G-Protein Coupled Receptor (GPCR) signaling. Not only are these models well-established; they are also biologically significant. In the case of EGFR signaling, this model represents dynamics upstream of the most well-studied pathway in cell signaling: MAPK [14]. In the second case, modeling GPCR signaling is important as GPCR’s are considered a prime drug target due to their ubiquity and targetability. In fact, around a third of drugs approved by the U.S. Food and Drug Administration (FDA) target this protein [15].

To understand what information is gained from using sequence or structure in the parameter inference of GPCR and EGFR signaling, we compare the results of two parameter inference approaches. In what we will refer to as the “baseline approach”, only the published data reported for the EGFR or GPCR model is used to infer model parameters. In the “augmented approach”, the reported data is used together with data from PDB or UniProt. Data from PDB and UniProt is integrated using our multiscale Bayesian framework. We compare the predictions and posterior inferred using the baseline approach to the predictions and posterior inferred using the augmented approach. Ultimately, we answer the question of what information can be gained from protein structure and amino acid sequence in the context of dynamic models of cell signaling.

## 2. Approach and results

### 2.1 Cell signaling ODE models

We base our investigation on two ODE models with different levels of complexity: a 50 parameter, 23 species model of EGFR signaling and an eight parameter, seven species model of GPCR signaling [16,17] (Table 1). In the case of EGFR signaling, a combination of mass action and Michaelis Menten kinetics is used to describe cell signaling events such as protein binding and phosphorylation. In the case of GPCR signaling, mass action kinetics are used to describe cell signaling events such as protein binding, activation, and degradation. Binding reactions are of particular interest to our study: in the case of EGFR, 18 parameters characterize nine binding reactions, while in the case of GPCR, two parameters characterize one binding reaction.

**Table 1:** ODE Model reaction list. Cyan, EGFR model; pink, GPCR model. Parameters for each reaction are included in parentheses to the right of each reaction. Ten binding reactions are bolded. Both models were previously published; the reaction lists are re-created using the respective publications [16,17]. Species abbreviations are consistent with previous publications, for clarity.

EGFR reactions		GPCR reactions
**R + EGF ⇌ R**_**a**_ **(k1f, k1b)**	**RP + Shc ⇌ R-Sh (k13f, k13b)**	**L + R ⇌ LR (k1f, k1b)**
R_a_ + R_a_ ⇌ R_2_ (k2f, k2b)	R-Sh ⇌ R-ShP (k14f, k14b)	Gd + Gbg → G (k2)
R_2_ ⇌ RP (k3f, k3b)	R-ShP ⇌ RP + ShP (k15f, k15b)	RL + G → RL + Ga + Gbg (k3)
RP → R_2_ (V4, K4)	ShP → ShC (V16, K16)	∅ ⇌ R (k4, k5)
**RP + PLC γ** **⇌ R-PL (k5f, k5b)**	**R-ShP + Grb ⇌ R-Sh-G (k17f, k17b)**	RL → ∅ (k6)
R-PL ⇌ R-PLP (k6f, k6b)	R-Sh-G ⇌ RP + ShG (k18f, k18b)	Ga → Gd (k7)
R-PLP ⇌ RP- PLCγP (k7f, k7b)	**R-Sh-G + SOS ⇌ R-Sh-G-S (k19f, k19b)**	
PLCγP → PLCγ (V8, K8)	R-Sh-G-S ⇌ Sh-G-S + RP (k20f, k20b)	
**RP + Grb ⇌ R-G (k9f, k9b)**	**ShP + Grb ⇌ Sh-G (k21f, k21b)**	
**R-G + SOS ⇌ R-G-S (k10f, k10b)**	ShG + SOS ⇌ Sh-G-S (k22f, k22b)	
R-G-S ⇌ RP + G-S (k11f, k11b)	Sh-G-S ⇌ ShP + G-S (k23f, k23b)	
**G-S ⇌ Grb + SOS (k12f, k12b)**	R-ShP + G-S ⇌ R-Sh-G-S (k24f, k24b)	
	PLCγP ⇌ PLCγP-I (k25f, k25b)	

### 2.2 Data augmentation

**Fig 2:**
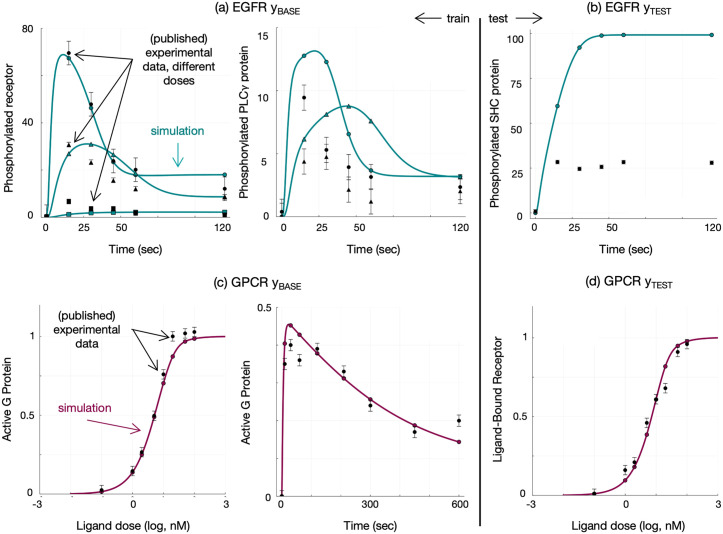
Baseline data set (${𝐲}_{BASE}$). Published measurements of in vitro protein dynamics for each ODE model. Eight EGFR outputs were observed and a representative sample of three are shown here. Three GPCR outputs were observed and all are shown here. (a) Training and (b) test data for EGFR inference. (c) Training and (d) test data for GPCR parameter inference. Cyan or pink lines, simulations using reported parameter values for EGFR and GPCR, respectively; black markers, reported experimental data points for a particular dose; black error bars, reported standard error.


Given an ODE model and experimental data, y, our objective is to infer the model parameters. In this work, we propose augmenting the published data for each system, $y_{BASE}$ (Fig 2), with predicted binding affinity values, ${\hat{K}}_{D}$. Equation (1) defines the augmented dataset, $y_{AUG}$,

$$\begin{array}{c} \begin{array}{c} \;y_{AUG}=\left[y_{BASE},\;{\hat{K}}_{D}\left(y_{PDB}\vee y_{FASTA}\right)\right] \end{array} \end{array}$$ (1)

We predict $K_{D}$ using experimental observations of amino acid sequences from UniProt, $y_{FASTA}$, or protein structures from PDB, $y_{PDB}$ (Table 2). Throughout the paper, we often refer to ${\hat{K}}_{D}(y_{PDB},y_{FASTA})$ as predicted $K_{D}$ for conciseness and clarity.

**Table 2:** Sequence (y_FASTA_) and structure (y_PDB_) data sets. Cyan, EGFR data; pink, GPCR data.

Signaling Molecule	System	Database	Database Identifier
y_PDB_			
GPCR bound to alpha pheromone	GPCR	Protein Data Bank	7QBI
y_FASTA_			
EGF	EGFR	UniProt	P01133
SHC1	EGFR	UniProt	P29353
PLCG1	EGFR	UniProt	P19174
SOS1	EGFR	UniProt	Q07889
GRB2	EGFR	UniProt	P62995
EGFR	EGFR	UniProt	P00533

To test our approach, we designate a portion of $y_{BASE}$ as test data for each ODE model. In the case of EGFR, the original work does not fit parameters to data, so we must decide our test/train data split. We choose to reserve measurements of one of the intracellular proteins, SHC, as our

test data. We choose SHC due to its clinical relevance, as this signaling species has been shown as an important marker in various cancers [18]. Furthermore, the published model predictions do not quantitatively match the experimental measurements of SHC [18]. This is apparent in Fig 2b. Ideally, incorporating data on protein structure will improve our predictions for this experimentally relevant target. Thus, we use the data for SHC to test our model predictions. For the GPCR model, as in the original work, we use the two G-protein data sets as training data. We reserve data on the bound receptor for testing.

Both ODE models also report binding parameter values derived from experiments or physical bounds, rather than fit to the data. We use these reported values as a test of our fitted forward and reverse binding rate constants, as well as to test our proposed $K_{D}$ predictor, detailed next.

### 2.3 Machine learning $K_{D}$ predictor pipeline

**Fig 3:**
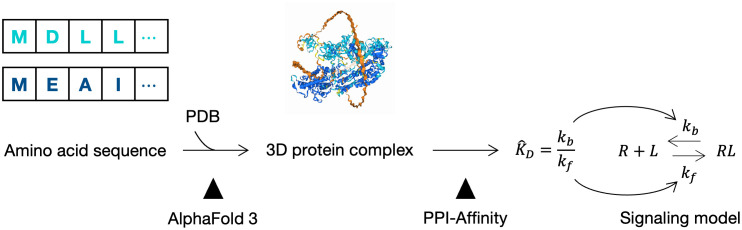
ML Pipeline to predict${𝐊}_{D}$. ${𝐊}_{D}$ is predicted using an SVM regression model, PPI-Affinity. PPI-Affinity needs the complex structure as an input. If the structure exists, it may be extracted from PDB. If it does not exist, the structure may instead be predicted using AlphaFold 3. AlphaFold 3 only needs amino acid sequences, which are typically available. The predicted $\begin{array}{c} {\text{K}}_{D} \end{array}$ is the same $\begin{array}{c} {\text{K}}_{D} \end{array}$ that characterizes reversible, bimolecular binding reactions comprising cell signaling models.

To leverage data on amino acid sequence and protein structure in the context of intracellular signaling models, we must translate the information in a sequence or a structure to a scale that is meaningful for cell signaling—that is, on the scale of protein concentrations. We developed a ML pipeline for this task (Fig 3).

Upon reviewing the literature, we found several ML models that predict protein binding affinity, $K_{D}$, in units of concentration, from structure or sequence. $K_{D}$ does not directly correspond to a parameter in dynamic ODE models. However, we can relate this predicted $K_{D}$ to the parameters of the ODE model, which are the unbinding ($k_{b}$) and binding ($k_{f}$) rates governing the reaction, by using the definition of $K_{D}$: the ratio of the unbinding rate to the binding rate. $K_{D}$ predictors are generally limited to bimolecular, reversible binding reactions. However, this is not a disqualifying limitation in the context of cell signaling models, which are often comprised of such reactions. This is because even when the product of the reaction contains more than two proteins, the process usually involves a series of bimolecular reactions [19].

We found two ML models with convenient webservers for making $K_{D}$ predictions: Prodigy and PPI-Affinity [20,21]. We moved forward with PPI-Affinity, a support vector machine (SVM) regression model, as it shows modest improvements on Prodigy’s performance. Further, it performs comparably to more complicated $K_{D}$ predictors, like those based on deep learning models [20]. Importantly, PPI-Affinity was benchmarked on several, curated protein-protein and protein-peptide binding datasets [22,23]. The $K_{D}$’s comprising these benchmarks span several orders of magnitude. The binding affinities we seek to predict were not included in these benchmarks, but their affinity magnitudes fall well within these benchmarks. This increases the likelihood that the EGFR and GPCR systems are within the applicability domain of applicability for PPI-Affinity while still providing new test data [24]. Further, PPI-Affinity provides both a protein-protein binding affinity predictor and protein-peptide binding affinity predictor. This is helpful, given our binding reactions comprise both protein-protein and protein-peptide binding.

PPI-Affinity requires the structure of the bound protein complex as an input; however, only a fraction of experimentally available protein structures are complexes. Instead, most structures available are for monomer proteins. Thus, we sought an alternative input to PPI-Affinity to use in cases where the protein structure is not available.

Excitingly, AlphaFold (AF3), the latest iteration of the deep learning model AlphaFold, is designed to predict the structures of biomolecular interactions, including protein complexes [25]. AF3 needs amino acid sequences of the binding partners to predict this structure. Fortunately, the amino acid sequence of a given protein of an intracellular signaling network is often known. AF3 is also hosted on a webserver, making predictions straightforward.

Altogether, we chain together existing ML models into one, user-friendly pipeline for predicting the binding affinity parameters of cell signaling models using amino acid sequence and protein structure.

### 2.4 Test case on uninformative prior

Before incorporating predictions from our ML pipeline into the parameter inference, we first tested its ability to accurately predict $K_{D}$ for the ten binding reactions present in the ODE test cases. For each of the ten binding reactions, we predict one $K_{D}$ using our proposed ML pipeline. This results in ten unique $K_{D}$ predictions. We compared the accuracy of these predictions to an uninformed $K_{D}$ prediction. To generate this uninformed prediction, we sampled our Bayesian prior ten times. This prior is log-uniform, and has been used as an uninformative distribution for parameter inference in systems biology [9]. Thus, it is well suited for representing an uninformed $K_{D}$ prediction. More details about this prior may be found in the Materials and Methods.

**Fig 4:**
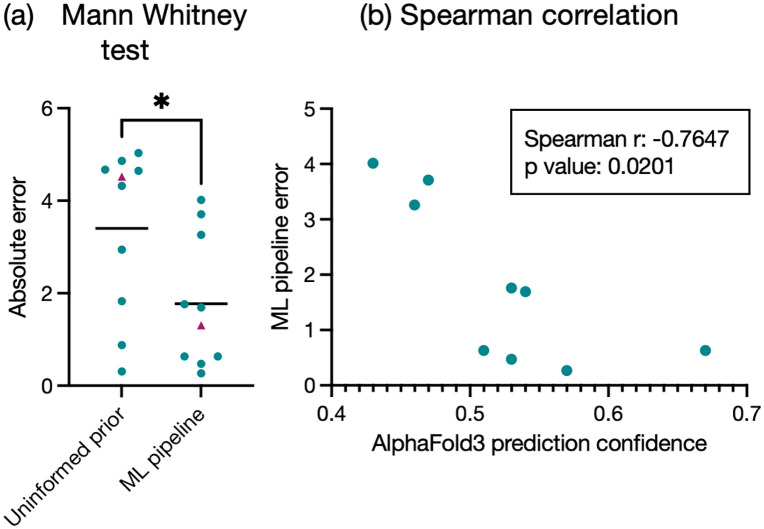
ML Pipeline performance. Cyan, EGFR reaction; pink, GPCR reaction. Circles, K_D_ predicted using predicted structure; triangles, K_D_ predicted using experimental structure. **(a)** Mann-Whitney test comparing absolute error of structure-informed K_D_ prediction with a random sample from a log-uniform distribution. n = 10 binding reactions. *p-value< 0.05. Black horizontal line, sample mean. **(b)** Spearman’s rank correlation between error of K_D_ prediction and AlphaFold 3 confidence metric, ranking score. n = 9 binding reactions that were predicted using a predicted structure, given there was no experimental structure.

We calculate the absolute error of the ML pipeline predictions and prior samples with respect to the experimentally-derived $K_{D}$ values reported in the GPCR and EGFR publications [16,17]. Since we are concerned with order-of-magnitude changes in $K_{D}$, this error is calculated after a log10 transformation of the ML pipeline prediction and reported $K_{D}$ values. Fig 4a shows how the ML pipeline’s performance compares to the uninformed prediction’s performance. The ML pipeline’s predictions have a significantly lower absolute error than the uninformed prediction. One of these $K_{D}$ predictions was made with an experimental structure. Interestingly, it resulted in only the fifth smallest error.

We also tested whether the confidence metrics provided by the ML models that comprise the pipeline were indicative of the pipeline’s overall performance. To do this, we calculated the Spearman correlation between the error of the ML pipeline’s prediction and each confidence metric—the applicability domain (AD) for PPI-Affinity and the ranking score for AlphaFold 3. More details on these metrics can be found in the Materials and Methods.

In the case of AF3, we test on only nine of the binding reactions. This is because for one of the reactions, there is an experimental structure. Thus, there was no need to use AlphaFold to predict the structure. We found that PPI-Affinity’s AD metric was not significantly associated with the overall pipeline’s performance (Fig A in S1 Text). However, we did find that AlphaFold 3’s ranking score was significantly correlated with the pipeline’s performance (Fig 4b).

### 2.5 Test case on EGFR and GPCR signaling: Parameters

**Fig 5:**
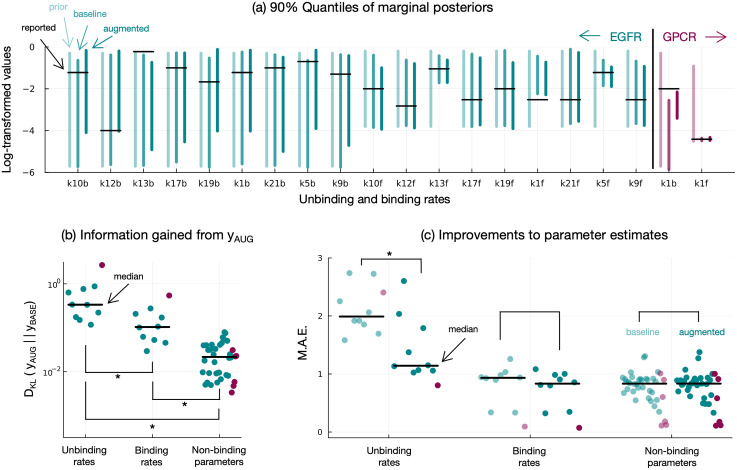
Parameter inference with and without data augmentation. Cyan, EGFR results; pink, GPCR results. All parameter samples were log-transformed prior to analysis. *p-value < 0.05 **(a)** 90% quantiles of marginal posterior distributions of binding parameters. Light colored line, prior; medium colored line, baseline posterior; dark colored line, augmented posterior; black horizontal line, reported parameter value. **(b)** KL divergence, in bits, from baseline posterior to augmented posterior. Values are grouped by parameter function. **(c)** Mean absolute error (M.A.E.) of parameter samples. Mean taken with respect to each posterior distribution. Error calculated with respect to the values reported in the literature. Light colored points, baseline; dark colored points, augmented; black horizontal line, median M.A.E.

Next, we infer the Bayesian posterior distributions of the ODE test model parameters using $y_{BASE}$ or $y_{AUG}$. Details on this inference can be found in the Materials and Methods. We analyze the posterior samples after a log10 transformation, as we want to compare order-of-magnitude changes in the parameters. The quantiles of the resulting marginal posterior distributions of the unbinding and binding rates are shown in Fig 5a. After checking that both inferences recapitulate the training data (Fig B in S1 Text), we analyzed the resulting posterior distributions. To measure the information gained from using the augmented dataset compared to the baseline dataset, we compute the KL divergence from the posterior estimated with $y_{BASE}$ to the posterior estimated with $y_{AUG}$. The resulting KL divergences are shown in Fig 5b. Interestingly, the most information is gained on the rate of protein unbinding. Fig 5c plots the mean absolute log error of the parameter estimates with and without data augmentation. Error is calculated with respect to the experimentally derived reported values from the published papers. Excitingly, we see that the information in $y_{AUG}$ improves the parameter estimates, especially in the case of the unbinding rates, where the improvement is statistically significant. This difference is significant for both a paired and unpaired t-test.

### 2.6 Test case on EGFR and GPCR signaling: Predictions

**Fig 6:**
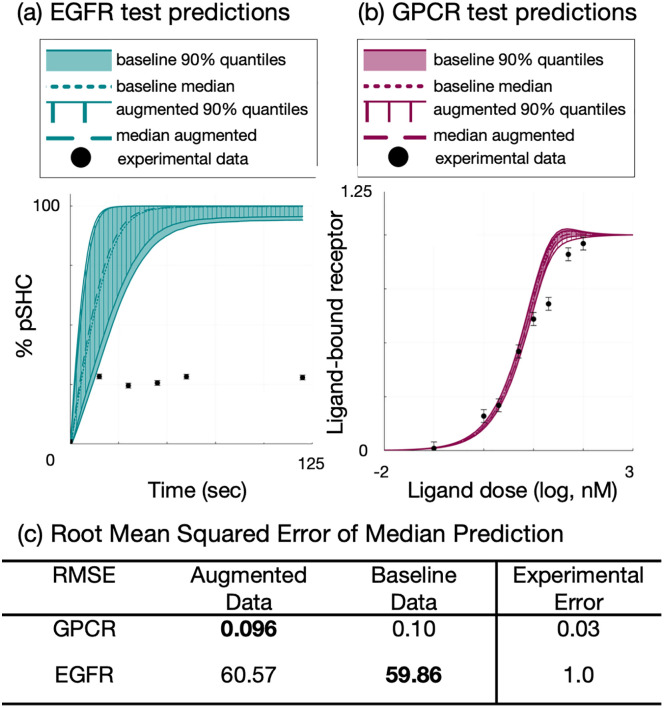
Performance on test set. **(a)** Predictions for EGFR test set, the percent of phosphorylated signaling protein SHC from 0-120 seconds. **(b)** Predictions for GPCR test set, the amount of ligand bound receptor 60 seconds post-stimulation, at different ligand doses, relative to 1000 nM of ligand. **(c)** Root mean squared error of median prediction. Cyan, EGFR; pink, GPCR. Shaded region, 90% quantiles of baseline approach; patterned region, 90% quantiles of augmented approach; dotted line, median prediction of augmented approach; dashed line, median prediction of augmented approach; black dots, experimental data with reported error.

Fig 6 shows the difference in the medians and 90% quantiles of the test predictions generated by sampling the augmented posterior compared to the baseline posterior. While we see some differences at middle timepoints of the EGFR test set (Fig 6a) and at certain doses of the GPCR test set (Fig 6b), overall the predictions are not qualitatively different. We also report the RMSE of the median predictions (Fig 6c). For both EGFR and GPCR, the difference between the RMSE of the augmented approach and baseline approach is not greater than the standard error reported for the experimental data. In other words, the difference in the performance between the two approaches falls within the error of the test set.

**Fig 7:**
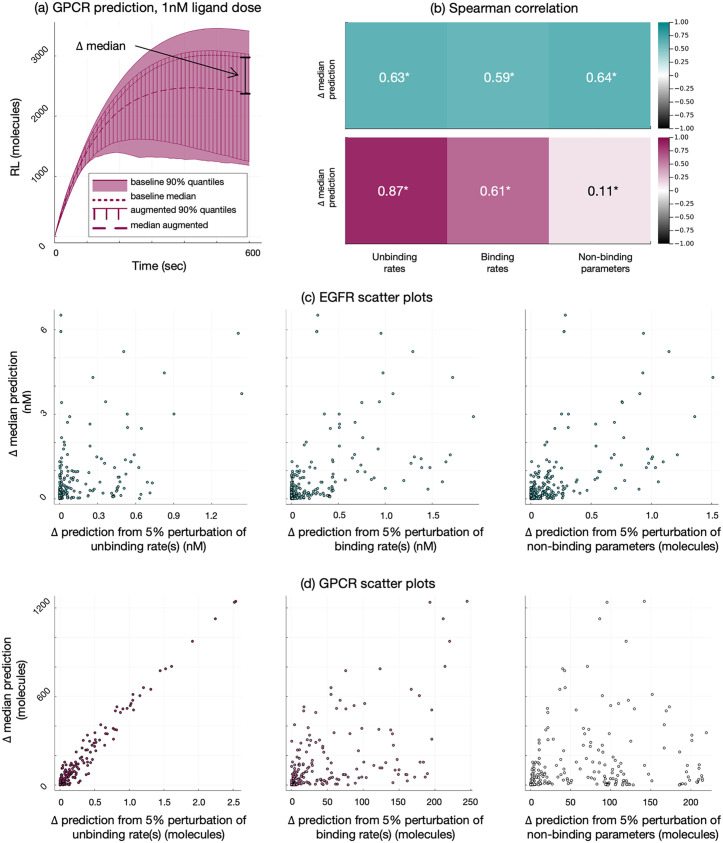
Local sensitivity analysis. **(a)** GPCR timeseries prediction of ligand-bound receptor, (RL), after 1 nM ligand stimulation. Units, molecules. Shaded region, 90% quantiles of baseline approach; patterned region, 90% quantiles of augmented approach; dotted line, median prediction of augmented approach; dashed line, median prediction of augmented approach. Difference between median prediction of baseline approach and median prediction of augmented approach, Δ median, indicated with black arrow. **(b)** Spearman correlation between Δ median and change in output due to 5% perturbation of either unbinding, binding, or non-binding parameters. Cyan, EGFR; pink, GPCR. *p-value< 0.05. n = 345 for EGFR; corresponding to 345 prediction outputs from 3 doses, 23 species, and 5 timepoints. n = 360 for GPCR; corresponding to 360 prediction outputs from 9 doses, 5 species, and 8 timepoints. Doses and timepoints match experimental observations. **(c)** Scatter plots illustrating relationship between perturbation and the Δ median prediction for GPCR. **(d)** Scatter plots illustrating relationship between perturbation to a type of parameter and the Δ median prediction for EGFR.

When we investigate the medians and 90% quantiles for other outputs besides the test set, we observe larger differences in the predictions. For example, if we consider the timeseries of receptor-bound ligand, RL, of GPCR at times other than 60 seconds post-stimulation, we see greater differences between both the quantiles and medians (Fig 7a). Similarly, several species comprising EGFR signaling show larger differences in the predicted median and 90 percent quantiles. (Fig C in S1 Text).

### 2.7 Robustness of results to changes in EGFR test/train split

While a test/train split was published with the original GPCR model, the EGFR model required we choose the test species—SHC. Other species could be held out from the parameter inference. To address this possibility, we exchanged the test species in a leave-one-out manner. The parameter estimation results were qualitatively and quantitatively consistent; KL divergence decreased from unbinding, to binding, to non-binding parameters and the M.A.E. improves significantly for unbinding parameters (Figs D-G in S1 Text). Similarly, the prediction performance results were quantitatively consistent; the difference between baseline RMSE and augmented RMSE was not larger than the reported experimental error (Fig H in S1 Text). However, the difference between the median baseline prediction and augmented baseline prediction varied, even becoming greater than the reported experimental error for the highest EGFR dose response. This result is consistent with an earlier observation; that distinct outputs are impacted differently by data augmentation. This result indicates that this observation is applies for predictions of experimental quantities as well.

### 2.8 Local sensitivity analysis

To investigate what leads to changes in some, but not all, ODE model predictions, we conducted a local perturbation analysis of both the GPCR and EGFR outputs to model parameters [2]. We perturbed the maximum likelihood parameter values of the baseline case by 5% and used the local sensitivity at the maximum likelihood to calculate the projected change in an output from this perturbation. More details about the local sensitivity analysis may be found in the Materials and Methods section.

Next, we calculated the Spearman correlation between the change in the median prediction of an output and the sensitivity of that output to perturbations of either unbinding rates, binding rates, or non-binding parameters. The resulting Spearman coefficients for both EGFR and GPCR are shown in Fig 7b. Both test cases display a positive and significant correlation with the sensitivity to perturbation of unbinding rates. In the case of GPCR, the correlation decreases from unbinding to binding to non-binding parameters. This decreasing correlation was clear visually (Fig 7c). Interestingly, this is the case despite the sensitivity to perturbations being around an order of magnitude lower for unbinding rates than the other parameter types. In the case of EGFR, there is a significant positive correlation for all parameter types.

### 2.9 Robustness of results to changes in prior distribution

The prior distribution for each binding parameter is representative of a non-informative characterization of our knowledge of binding and unbinding rates. However, we also wanted to explore how the results might change given a more informative or less informative prior. To do so, we increased or decreased the upper and lower bounds of the prior distributions of all parameters by one order of magnitude. The results were qualitatively consistent across these changes in the prior (Figs I-L in S1 Text). Further, they were mostly quantitatively consistent as well; only a couple quantitative differences emerged. In the case of the more informative prior, the difference between median predictions for the EGFR test data was no longer greater than the reported error at any individual point (Fig J in S1 Text). In the case of the less informative prior, the information gained from data augmentation was no longer significantly different between the unbinding and binding rates (Fig K in S1 Text). This was because more information was gained for binding rates. Interestingly, this did not result in a corresponding significant change in the mean absolute error for binding rates.

## 3. Discussion

In this work, we explored the potential of augmenting the conventional measurements used for parameter inference in models of intracellular signaling. Ultimately, our goal was to reduce uncertainty in parameters for better model determination. To do this, we proposed a multiscale, Bayesian modeling framework. We first develop a ML pipeline for incorporating the new, multiscale measurements into parameter inference. Then, we conduct a thorough quantification of the information gained from these new measurements using a Bayesian parameter inference.

We use amino acid sequence and protein structure measurements from UniProt and PDB, respectively, to augment the published datasets of two test cases: EGFR and GPCR signaling. The published datasets include partial, relative measurements of protein concentration and protein co-localization over time. We chose the EGFR and GPCR models as our test cases because both are well-established and biologically significant. Taken together, these models offer a comprehensive test of our approach. For example, EGFR provides a test of a moderately sized model, while GPCR provides a test of a minimal model. EGFR provides a test of predictions using sequence, while GPCR provides a test of predictions using structure.

Predictions from the GPCR and EGFR models occur on the scale of protein concentrations. To make measurements of sequence and structure applicable to the parameter estimation of these models, we needed to convert the measurements to the same scale as the GPCR and EGFR models. Excitingly, we were able to integrate two ML models, PPI-Affinity and AlphaFold 3, into one pipeline to do this [20,25]. This pipeline uses sequence or structure to predict the binding affinity, $K_{D}$, of a given bimolecular, reversible reaction in a cell signaling model. We find that this ML pipeline outperforms an uninformative prior when predicting the $K_{D}$. While these predictions do demonstrate noticeable variability, there is nonetheless a significant improvement in the average absolute error. Thus, it is appropriate to use when no experimental measurements exist for $K_{D}$. Importantly, this pipeline is generalizable to other dynamic signaling models. It is limited only by access to amino acid sequence and comprises two, user-friendly web servers.

Interestingly, we found that our pipeline’s performance did not significantly benefit from using an experimental protein complex structure, rather than predicted complex structure, when predicting $K_{D}$. The binding affinity between GPCR and its ligand was predicted using an experimental structure. However, the error of this prediction was not smaller than all the other $K_{D}$ prediction errors. PPI-Affinity was trained using complex structure and $K_{D}\;$pairs from the PDBbind database. Although the GPCR structure was within the applicability domain of PPI-Affinity, it seems that the ligand-bound GPCR complex was not included in this training set. This could explain the lack of improvement we see. We also note the small sample size (n = 1) of experimental structures we tested, which limits the certainty of this result.

On the other hand, there was a significant negative correlation between AlphaFold 3’s confidence metric and the error of the $K_{D}$ prediction. An interesting future direction could be investigating other probabilistic frameworks for incorporating AlphaFold’s confidence into parameter inference. For example, a power prior; power priors consist of raising the likelihood, or a term in the likelihood, to a discounting factor. This factor controls the amount of information taken from certain data [26,27]. Such a discounting factor could be derived from AlphaFold’s confidence, effectively down-weighing low-confidence predictions.

We integrate the predicted $K_{D}$’s into the likelihood function of our signaling model’s parameter inference. We combined this likelihood of the predicted $K_{D}$ and the likelihood of the data reported in the original publications to form an augmented dataset, y_AUG_. This augmented dataset is ultimately informed by the experimental data on sequence or structure that we use to predict $K_{D}$. We then compared the posteriors and predictions inferred with y_AUG_ to the posteriors and predictions inferred using only the original data, y_BASE_, our baseline dataset.

Overall, augmenting with data on sequence or structure provides more information on rates of protein unbinding compared to using only the original data. This was consistent across both the EGFR test case and GPCR test case. Further, the information gained from sequence or structure tends to push the unbinding rate towards the rates reported in the original publications. These reported rates were all taken from experiments, rather than fit to data [16,17]. Our results offer an understanding of when our approach is useful: in the case of underdetermined rates of protein unbinding. We have confidence in this recommendation of utility. First, because the results were consistent even with a more informative or less informative prior distribution. Second, because the results were consistent even with a different test/train division for the ODE model parameter inference. In practice, it is helpful to use our approach to constrain unbinding rates, as these rates tend to vary over a larger range than binding rates [19].

When we investigate how predictions change due to the data augmentation, the results are less clear. We first find that augmenting the baseline data set does not fundamentally change the predictions of our test data for either test case. However, we do see more substantial changes for other outputs besides the test data. We thus investigated when predictions inferred with $y_{BASE}$ will differ from predictions inferred with $y_{AUG}$. As our approach changes unbinding parameter rates, intuitively, one would then expect to see differences in predictions for outputs influenced by unbinding rates. Thus, we investigated the relationship between the local sensitivity of an output to unbinding rates, binding rates, and non-binding parameters. When we analyzed the GPCR model, there emerged a striking, significant, positive and linear correlation between changes in a prediction and the local sensitivity of that prediction to perturbations in the unbinding rate. A significant positive correlation also emerged with the EGFR model. These sensitivity analyses provide a key insight into the utility of this approach; augmenting data is most useful for influencing predictions that are sensitive to unbinding rates.

Interestingly, the magnitude of the correlation between the local sensitivity and differences in predictions was similar across unbinding rates, binding rates, and nonbinding parameters for the EGFR model. The different results from the two test cases may be a function of the complexity of the models. While GPCR is a minimal model comprised of eight parameters, EGFR is a more moderately sized model comprised of 50 parameters. It is thus plausible that the relationship between sensitivity and predictions is impacted by higher-order interactions between parameters in the case of EGFR [28]. These interactions would not be captured by the local sensitivity analysis we conduct here.

We acknowledge some limitations of our study. First, it is important to note that intracellular signaling, the biological system of interest here, poses unique challenges in protein structure prediction. Our ML pipeline predicts the binding affinities of bi-molecular binding reactions in signaling networks. Binding reactions in the context of cell signaling can involve conformation selection and/or induced-fit mechanisms upon complex formation. Such binding mechanisms can pose a challenge for prediction via machine learning models like AlphaFold [25,29]. AlphaFold3 predicts one, static protein conformation. Especially in the case of flexible proteins like GPCR and EGFR, this can limit the predictive power of binding affinity models, like PPI Affinity, that require the binding-competent state for accurate prediction [30]. While our results establish the potential of even static protein conformation simulations, a valuable future study could investigate the robustness of the binding affinity predictor to protein conformation changes. This could be done using molecular dynamics simulation.

Second, we test our ML pipeline on only the 10 binding reactions reported in our test cases. In the future, we could test our pipeline with more reactions from databases of paired structures and affinities, like PDBbind [23]. Such an expanded test sample could help benchmark our approach’s performance on more difficult or out-of-distribution predictions than the ones we test here.

Finally, our approach does not predict ODE model kinetic off and on rates directly. Predicting the ODE model parameters directly would be preferable to predicting the ratio of parameters ($K_{D}$). However, in contrast to binding affinity, the prediction of kinetic off and on rates has received considerably less attention in the computational chemistry field [31]. Thus, when we evaluated existing models for predicting binding rates, we found them to be less generalizable than $K_{D}$ predictors. In future, improved binding rate predictors could be used to predict ODE model parameters directly. Our Bayesian framework can evolve with such improved predictors. For example, we use the error reported by PPI-Affinity to weigh the predicted $K_{D}$ likelihood against the likelihood of the conventional experimental measurements. This weight can be adjusted as that error improves.

There are also ML models that use amino acid sequence to predict binding affinity. In theory, we could use one of these models instead of our pipeline. However, in practice, we found that there were no such models hosted on convenient webservers. Even more limiting was the prohibitively large RAM these models needed to make a prediction for the relatively large signaling protein complexes we are interested in here [32]. We instead focus on more user-friendly models, both in terms of RAM and implementation.

In conclusion, we have developed a multiscale, Bayesian framework to show that it is viable to use measurements of amino acid sequence and protein structure to augment parameter inference in the context of dynamic signaling models. Experimental data on protein structure and amino acid sequence consistently adds information on the unbinding rate parameters of these signaling models. This improves parameter estimation and thus model determination. In the future, our framework has the potential to synergize with breakthroughs in ML and data collection. As ML models better predict binding affinities, our framework can better constrain signaling models. As databases of protein structure and amino acid sequence grow, our framework will generalize to more binding reactions. Ultimately, this synergy can enable more biologically-grounded simulations by bridging data on sequence and structure with systems level signaling.

## 4. Materials and methods

### 4.1 ODE model simulation

We simulate both models with the Julia package *DifferentialEquations.jl* [33]. The EGFR model simulates signaling up to 120 seconds, while the GPCR model simulates signaling up to 600 seconds. We use the reported initial species pools to initialize the model simulations.

Because our model parameter values can vary across a wide range, the solution may be stiff. Thus, we want to choose an ODE solver that can handle stiffness. In addition, we need a solver that performs well given a higher-than-default accuracy requirement. This is due to the nature of our state variable: protein concentration. The accuracy of a particular problem is specified by the absolute and relative tolerances. The relative tolerance (rtol) is set based on the number of significant digits required for the model output, plus 1 [34,35]. We note that the upper bound on the number of significant digits is between 15 and 17, as this is the maximum number of significant digits stored by a Float64 type variable, which we use here. In our case, we deem 5 significant digits sufficient for our problem and set the rtol to 10^-(5 + 1)^. The absolute tolerance (atol) is set based on the absolute value of the model output that may be deemed insignificant [34,35]. In our case, we deem a protein concentration of 10^-5^ nM, or less than 1 protein/cell, as insignificant. Reassuringly, a rtol of 10^-6^ and atol of 10^-5^ are comparable to the default tolerances for most scientific computation, 10^-3^ and 10^-6^, respectively [34,33]. Based on our requirements, we choose to use *DifferentialEquation.jl*’s QNDF solver, which is equivalent to Matlab’s ode15s solver [33]. It is a stiff solver that can handle medium tolerances; thus, it meets our problem’s requirements [34,33,36].

### 4.2 Extracting experimental measurements of EGFR and GPCR signaling

Experimental measurements for the ODE models are reported as plots. We extracted the data and errors with Plot Digitizer [37].

### 4.3 Extracting measurements of EGFR and GPCR sequence or structure

To find relevant protein complex structures, we searched the Protein Data Bank by macromolecule, filtering out complexes that comprised either non-natural amino acids—like structure 5CZI which contained a phosphotyrosine—or fragments of the complete structure—like structures 1GBR and 1QG1. The former is filtered out because PPI Affinity cannot generate feature vectors for non-natural amino acids. The latter is filtered out because the full complex has been shown to provide critical context for predicting binding affinity [38,39]. We also checked that including fragments would worsen our ML pipeline performance; we found that, indeed, when we used complexes comprising fragments to make predictions, the ML pipeline did not perform significantly better than an uninformed prior (Fig M in S1 Text).

When protein complex structures were not available, we pulled the canonical sequence of each protein in the complex from the Universal Protein Resource (UniProt).

### 4.4 Implementation of ML pipeline to predict $K_{D}$

Fig 3 provides an overview of the ML pipeline used to predict $K_{D}$ using amino acid sequence ($y_{FASTA}$) or protein structure ($y_{PDB}$). Given either the sequences of the binding proteins or the structure of the bound complex, our objective is to predict the binding affinity, $K_{D}$, in units of concentration, for a specific bimolecular binding reaction. We can then relate this predicted $K_{D}$ to the parameters of the ODE model, which are the unbinding ($k_{b}$) and binding ($k_{f}$) rates governing this reaction, by using the definition of $K_{D}$: the ratio of the unbinding rate to the binding rate.

If the bound complex structure is available, we can use a support vector machine (SVM) regression model, PPI-Affinity, to predict ΔG, the free energy change in kcal/mol [20]. PPI-Affinity uses a feature vector derived from the complex structure and polynomial function kernel to make its prediction of ΔG. We then convert ΔG to $K_{D}$, given a gas constant and the environment’s absolute temperature [22,40]. If the bound complex is not available, we use a deep-learning model, AlphaFold 3, to predict the complex structure given the amino acid sequences of the individual proteins. If the complex is comprised of identical subunits, we extract the smallest subunit [41]. AlphaFold 3 provides 5 replicates per structural predictions—each with a different initial seed. Given the significant correlation between pipeline performance and AlphaFold3’s confidence, we use the highest-confidence replicate as in input to PPI-Affinity to predict $K_{D}$.

Both PPI-Affinity and AlphaFold 3 provide confidence metrics for their predictions. AlphaFold provides a ‘ranking score’ for each predicted structure. This score ranges from [-100, 1.5] with -100 representing low confidence. The ranking score is a function of various features of the protein structure, such as the fraction of the predicted structure that is disordered and the interface predicted template modeling (ipTM) score [25]. Both the ranking score and ipTM are significantly correlated with the performance of the ML pipeline (Figs 4b and A in S1 Text). Conveniently, ipTM is scaled between [0,1], with zero representing low confidence. In the case of PPI-Affinity, it is noted whether each $K_{D}$ prediction is within the applicability domain of the training dataset [20]. We found that the accuracy of predictions was more dependent on ranking score than applicability domain.

### 4.5 Bayesian inference

The objective of Bayesian inference is to find the posterior distribution, p(θ|y) , which is defined using Bayes rule:


$$\begin{array}{c} p\left(\theta |y\right)=\;\frac{p\left(y|\theta \right)p(\theta )}{p(y)} \end{array}$$

While we often know the likelihood of the data given model parameters, p(y|θ), and the prior probability of parameters, p(θ) we usually do not know the probability of the data p(y). Thus, we must approximate this distribution knowing p(θ|y)  up to a proportionality constant. While there are various distribution approximation methods for this problem, the most powerful are Markov-Chain Monte Carlo (MCMC) approximations, because our initial guess of p(θ|y) does not need to be particularly close to the reality. These algorithms generate correlated samples of the posterior, which we may use to simulate our model.

To generate these samples for our ODE models, we use a Julia implementation of the affine invariant ensemble MCMC sampler from the package *AffineInvariantMCMC.jl* [42]. Like all MCMC algorithms, the affine-invariant ensemble sampler algorithm constructs a Markov chain that is used to sample the posterior distribution. To better navigate the parameter space, this algorithm initializes an ensemble of samplers that propose the next sample based on the location of another sampler in the ensemble. Because all our parameter values are restricted to positive values and are often described on a logscale rather than linear scale, we conduct sampling in a log-transformed parameter space [43,44]. Further discussion of the convergence diagnostics of the samples to the posterior may be found in Section 1.1 in S1 Text.

### 4.6 Prior

The prior encodes our assumptions, ideally derived from empirical knowledge, about parameters. Here, we choose to define independent, log-uniform priors. If the parameter is a binding rate, we limit the distribution to [10^-4^ nM^-1^s^-1^, 10^0^ nM^-1^s^-1^]; for an unbinding rate, we limit the distribution to [10^-6^ s^-1^, 10^0^ s^-1^]. This is in line with prior distributions for binding rates in the literature and database values [9,45]. The unbinding rate prior is wider than the binding rate prior, which is also in line with the literature [19]. For non-binding parameters, we limit the distributions to +/- two orders of magnitude above and below the reported parameter value. Specific details on the prior distribution are included in Table A in S1 Text.

### 4.7 Likelihood

Recall that our augmented data, y, is comprised of two categories, $y_{BASE}$ and predicted $K_{D},$ ${\hat{K}}_{D}$. Both $y_{BASE}$ and ${\hat{K}}_{D}$ represent vectors of data points. For example, in the case of EGFR, $y_{BASE}$ is a vector of 42 points, corresponding to seven different outputs observed at six timepoints each, while ${\hat{K}}_{D}$ is a vector of nine predicted affinities for the nine binding reactions of the model.

We assume that each data point is independent from the rest. Next, we assume that a data point in $y_{BASE}$, $\overset{j}{\underset{BASE}{y}},\;$ is normally-distributed, given the ODE model prediction and experimental error for data point j. For example, if j indicates the amount of active G-protein 300 seconds after stimulation, then we use the model’s prediction for active G-protein 300 seconds after stimulation as the mean of the likelihood distribution and the reported error of this measurement as the deviation of the likelihood distribution.

Finally, we assume that a data point in ${\hat{K}}_{D}$, $\overset{k}{\underset{D}{\hat{K}}}$, is log-normally distributed, given the ratio of the unbinding and binding reaction rates for that data point and standard error of the SVM regression model, PPI-Affinity. For example, if $\overset{k}{\underset{D}{\hat{K}}}$ is the predicted binding affinity of a receptor binding a ligand, then we will use the rate of receptor ligand unbinding divided by rate of receptor ligand binding as the mean of the likelihood distribution and the standard error as the deviation. This standard error is on the scale of one order of magnitude change in ${\hat{K}}_{D}$. Further details on our likelihood distribution are included in Section 1.3 and Table A in S1 Text.

### 4.8 Log transformation of parameters for analysis

We note that analyses of the model parameters, and therefore posteriors, are done after a log10-transformation of the parameter samples. The reason for this is threefold. First, log-transformation tends to bring biochemical parameters like $K_{D}$ towards a Normal distribution, which is desirable for most statistical analyses [43]. Second, by comparing order-of-magnitude changes in parameters, we can compare parameters of different scales. Finally, we are concerned with order-of-magnitude changes in $K_{D}$, as it has been shown that changes less than this do not significantly influence model predictions [46].

### 4.9 Quantifying information gained using KL divergence

We use KL divergence between marginal posterior distributions to quantify the information gained from augmenting our data with experimental measurements of amino acid sequence and protein structure. Specifically, we calculate the KL divergence from the marginal posterior of parameter $\theta_{i}$ given only the baseline data, $q(\theta_{i}|y_{BASE})$, to the marginal posterior of $\theta_{i}$ estimated with the augmented data, $p(\theta_{i}|y_{AUG})$:


$$\begin{array}{c} D_{KL}(P||Q)=\;\int_{\text{0}}^{\infty }p(\theta_{i}|y_{AUG})\;\text{log}\frac{p(\theta_{i}|y_{AUG}):\;}{q(\theta_{i}|y_{BASE})} \end{array}$$

Given posterior samples, we use a univariate kernel density estimate and Monte Carlo integration to approximate a divergence for each marginal posterior [12].

### 4.10 Sensitivity analysis and perturbation analysis

We compute the local sensitivity of a model prediction with respect to model parameters using Julia’s *ForwardDiff.jl* packages [47–49]. Given the local nature of this analysis, parameters are not log-transformed. *ForwardDiff.jl* approximates the derivative of an output with respect to a model parameter using forward mode automatic differentiation. We use forward mode automatic differentiation as it is recommended for ODE’s with less than 100 parameters [47]. We take the local sensitivity with respect to the posterior parameter set that maximizes the likelihood of the baseline training data.

To calculate the projected change in an output due to a 5% change in a particular parameter, we multiple the local sensitivity by 5% of the maximum likelihood parameters [2]. We take the absolute value of the resulting perturbation. We then need to down select one perturbation value for each type of parameter: unbinding, binding, or non-binding. In the case of GPCR, this is trivial for unbinding and binding rates, as there is only one unbinding parameter and one binding parameter. For the other cases, GPCR non-binding parameters and all EGFR parameters, we take the maximum resulting perturbation.

### 4.11 Statistical analyses

All statistical analyses, Mann Whitney U test, Wilcoxon signed rank test, and Spearman rank correlation, were carried out using Prism 10.4.0 or a combination of Julia’s *HypothesisTests.jl* package and *StatsBase.jl* packages.

Given the small sample size, we used an exact Mann Whitney U test and Wilcoxon signed rank test.

## Supporting information

S1 Text Supplementary material. Supplementary Table and Figures, along with Supplementary Methods with details on Bayesian Inference.
(PDF)
