# Supplementary material for: A multiscale, Bayesian inference approach to augment mechanistic models of cell signaling with machine-learning predictions of binding affinity
Source: PLoS Comput Biol. 2026 Jun 5;22(6):e1014321. doi: 10.1371/journal.pcbi.1014321 (PMC13258154; doi:10.1371/journal.pcbi.1014321)
Supplement: S1 Text — Supplementary Table and Figures, along with Supplementary Methods with details on Bayesian Inference. (PDF) [file pcbi.1014321.s001.pdf]

Multiscale Probabilistic Modeling: A Bayesian Approach to Augment Mechanistic Models of Cell Signaling with Machine-Learning Predictions of Binding Affinity

Holly A. Huber[a] and Stacey D. Finley[a,b,c]

[a]Alfred E. Mann Department of Biomedical Engineering, University of Southern California, Los Angeles, California , United States of America

[b]Department of Quantitative and Computational Biology, University of Southern California, Los Angeles, California, United States of America.

[c]Mork Family Department of Chemical Engineering and Materials Science, University of Southern California, Los Angeles, California, United States of America

SUPPLEMENTARY INFORMATION

1. Supplementary Methods – Details on Bayesian Inference

We use 1000 walkers to sample the 8-parameter GPCR model and 50-parameter EGFR model. This was a conservative number, chosen to exceed the minimum number of walkers for sampling, which is 2*number of parameters (1). These walkers are dependent, thus traditional metrics comparing variance within and between chains are not applicable (2).

1.1 Convergence Diagnostics

We use the effective sample size, ESS, to monitor convergence to the posterior. ESS is a function of the autocorrelation of samples generated by the MCMC algorithm. Monitoring autocorrelation is the recommended metric for the affine invariant sampler, thus, we use ESS here (2). We conclude convergence once there are 100 effective samples per ensemble chain (3).

1.2 Parameter Distributions

Table A. Parameters defining likelihood and prior distributions.

**Likelihood of Experimental Data Point *j***	**Likelihood of Experimental Data Vector**
$x_j \vert \vec{\theta} \sim \mathcal{N}\left(\mathcal{M}(\vec{\theta})_j, {\sigma_j}^2\right)$	$\vec{x} \vert \vec{\theta} \sim \prod_j \mathcal{N}\left(\mathcal{M}(\vec{\theta})_j, {\sigma_j}^2\right)$
${\sigma_j}^2 = \text{experimental error reported for data point j}$<br>$x_j = \text{experimental data point j}$<br>$\vec{\theta} = \text{vector of ODE model parameters}$<br>$\vec{x} = \text{vector of experimental data}$<br>$\mathcal{M}(\vec{\theta})_j = \text{ODE model prediction for data point j, evaluated with parameter vector } \vec{\theta}$	
**Likelihood of Predicted Binding Affinity, $\widehat{K}_D$, for Binding Reaction *i***	**Likelihood of $\widehat{K}_D$ Vector**
$\log \widehat{K}_{D,i} \vert \vec{\theta}_i \sim \mathcal{N}\left(\log \frac{\theta_{\text{off},i}}{\theta_{\text{on},i}}, \sigma^2\right)$	$\overrightarrow{\log\ \widehat{K}_D} \vert \vec{\theta} \sim \prod_i \mathcal{N}\left(\log \frac{\theta_{\text{off},i}}{\theta_{\text{on},i}}, \sigma^2\right)$
$\widehat{K}_{D,i} = \text{ML pipeline prediction of binding affinity for reaction } i$<br>$\vec{\theta}_i = (\theta_{on,i}, \theta_{off,i}) = \text{ forward and reverse binding parameters for reaction } i$<br>$\sigma^2 = \text{order} - \text{of} - \text{magnitude error reported for ML pipeline, see next section for details}$	
**Prior Distribution of Parameter *k***	**Prior Distribution of Parameter Vector**
$\log \theta_k \sim U(\log lb_k, \log ub_k)$	$\overrightarrow{\log \theta} \sim \prod_k U(\log lb_k, \log ub_k)$
$lb_k = \text{lower bound for parameter } k$<br>$ub_k = \text{upper bound for parameter } k$	

1.3 K_D Likelihood Standard Deviation

We use the M.A.E. reported by PPI Affinity as the standard deviation for the augmented likelihood term. PPI Affinity reports the mean absolute errors, M.A.E., in kcal/mol, for two test sets. One test set is comprised of protein-peptide binding reactions. The M.A.E. for this set is 1.1 kcal/mol. Another test set is comprised of protein-protein binding reactions. The M.A.E. for this set is 1.8 kcal/mol (4). We use the first M.A.E. as the standard deviation for the likelihood of

K_Ds characterizing protein-peptide binding reactions and the second M.A.E. as the standard deviation for the likelihood of K_Ds characterizing protein-protein binding reactions.

To use the reported M.A.E., we need to convert from a change in energy in kcal/mol to a change in the binding affinity. A change in energy of about 1.35 kcal/mol results in an order of magnitude, or 10x, change in the binding affinity at room temperature (5). However, we are concerned with changes at body temperature—in that case, a change in energy of about 1.41 kcal/mol results in an order of magnitude change in K_D. Using this relationship, we calculate the order of magnitude change in K_D given the changes in energy reported by PPI Affinity:

$$\frac{1}{1.41} = \frac{\sigma_{pep-prot}}{1.1} => \sigma_{pep-prot} = 0.8$$

$$\frac{1}{1.41} = \frac{\sigma_{prot-prot}}{1.8} => \sigma_{prot-prot} = 1.3$$

The augmented likelihood is on a log 10 scale. Thus, there are no further conversions needed to use these values in our likelihood. Overall, this means that we penalize the augmented likelihood term on the scale of one order of magnitude change in K_D—slightly less in the case of a peptide-protein interaction and slightly more in the case of a protein-protein interaction.

2. Supplementary Figures

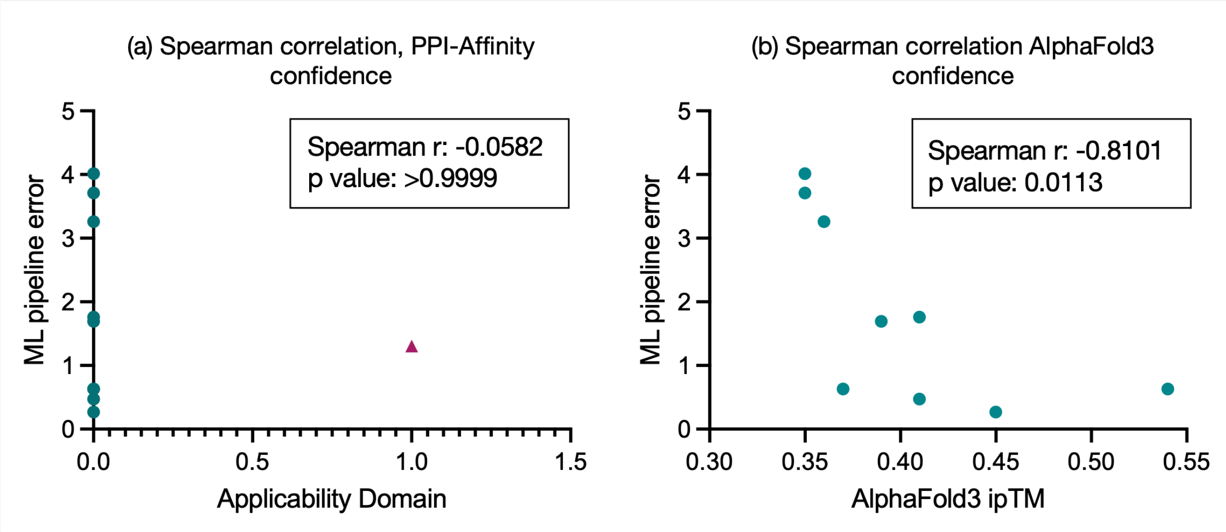

Fig A. ML Pipeline extended performance. Cyan, EGFR reaction; pink, GPCR reaction. Circle, predicted structure, triangle, experimental structure. (a) Spearman's rank correlation between error of K_D prediction and PPI-Affinity confidence metric, Applicability Domain. Applicability Domain is a binary metric, with 1 indicating confidence. n = 10 binding reactions. (b) Spearman's rank correlation between error of K_D prediction and AlphaFold 3 confidence metric, ipTM. ipTM bounded from [0,1]. n=9 binding reactions for which we used predicted structure, given there was no experimental structure.

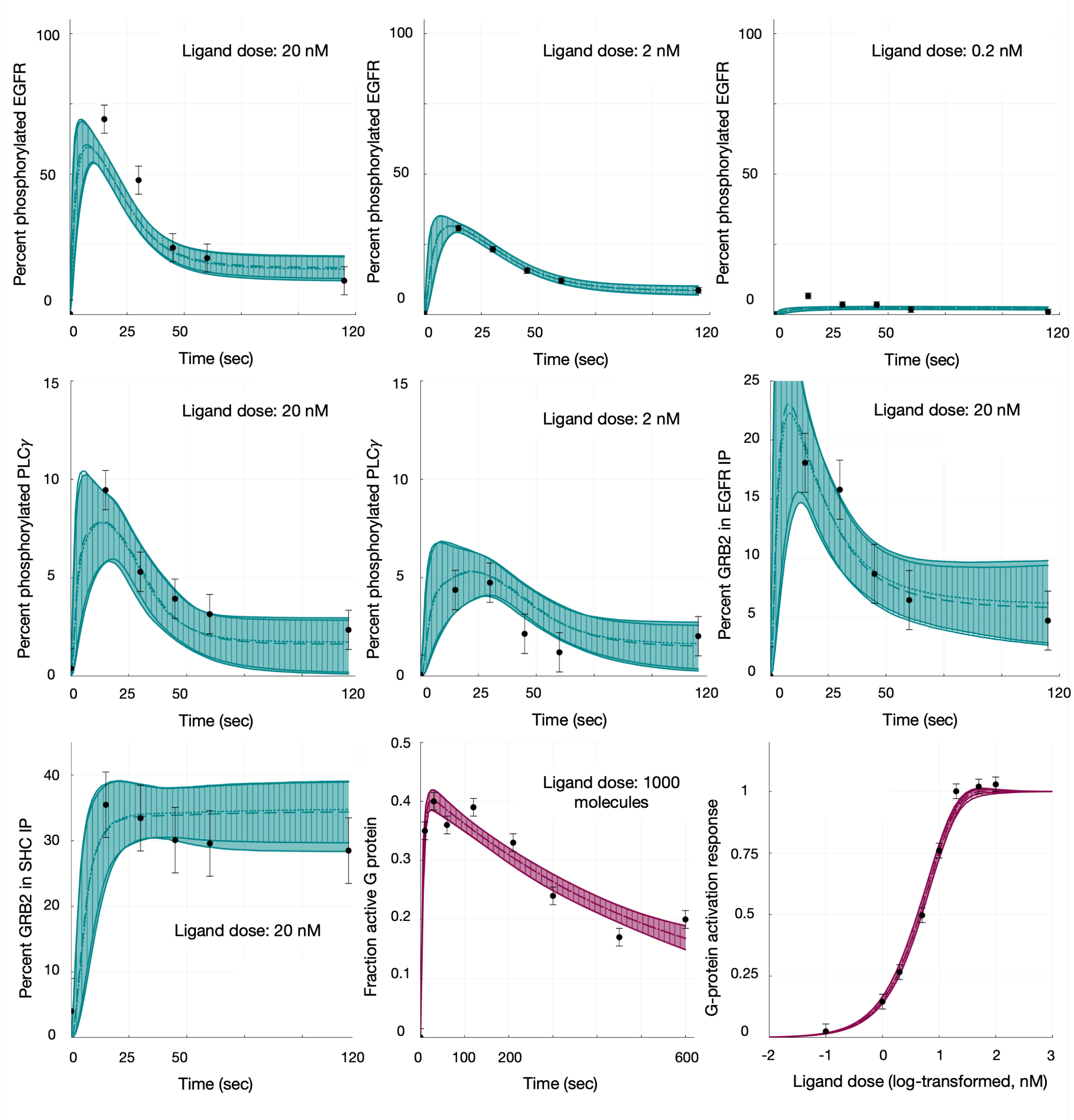

Fig B. Training data performance. Cyan, EGFR; pink, GPCR. Shaded region, 90% quantiles of baseline approach; patterned region, 90% quantiles of augmented approach; dotted line, median prediction of augmented approach; dashed line, median prediction of augmented approach; black dots, experimental data with reported error.

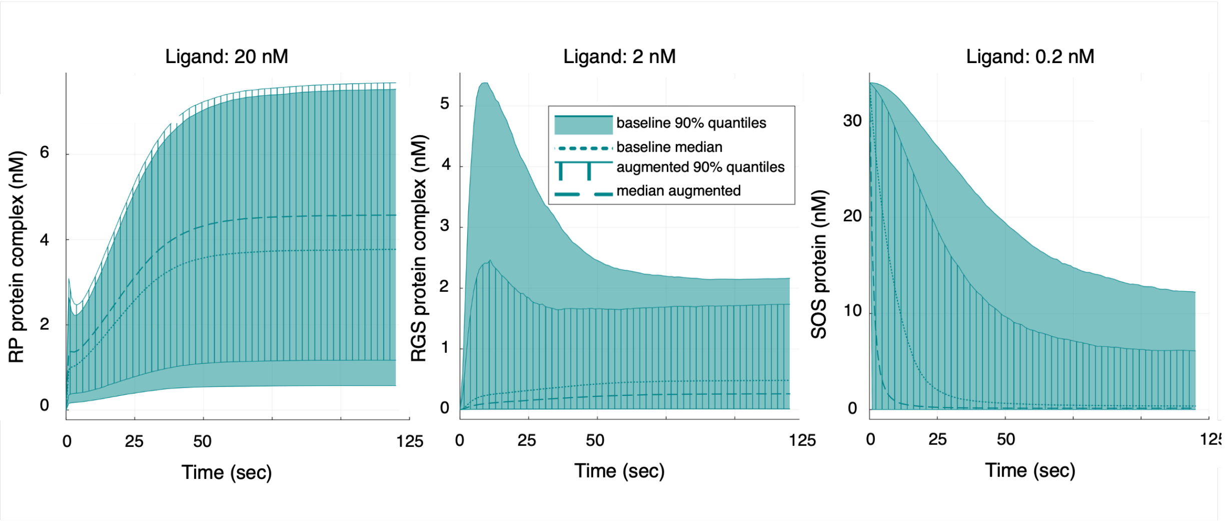

Fig C. EGFR timeseries predictions across time, ligand dose, and species. RP, phosphorylated, ligand-bound receptor; RGS, phosphorylated, ligand-bound receptor bound to intracellular proteins GRB2 (G) and SOS (S). Prediction in nanomolar concentration. Shaded region, 90% quantiles of baseline approach; patterned region, 90% quantiles of augmented approach; dotted line, median prediction of augmented approach; dashed line, median prediction of augmented approach.

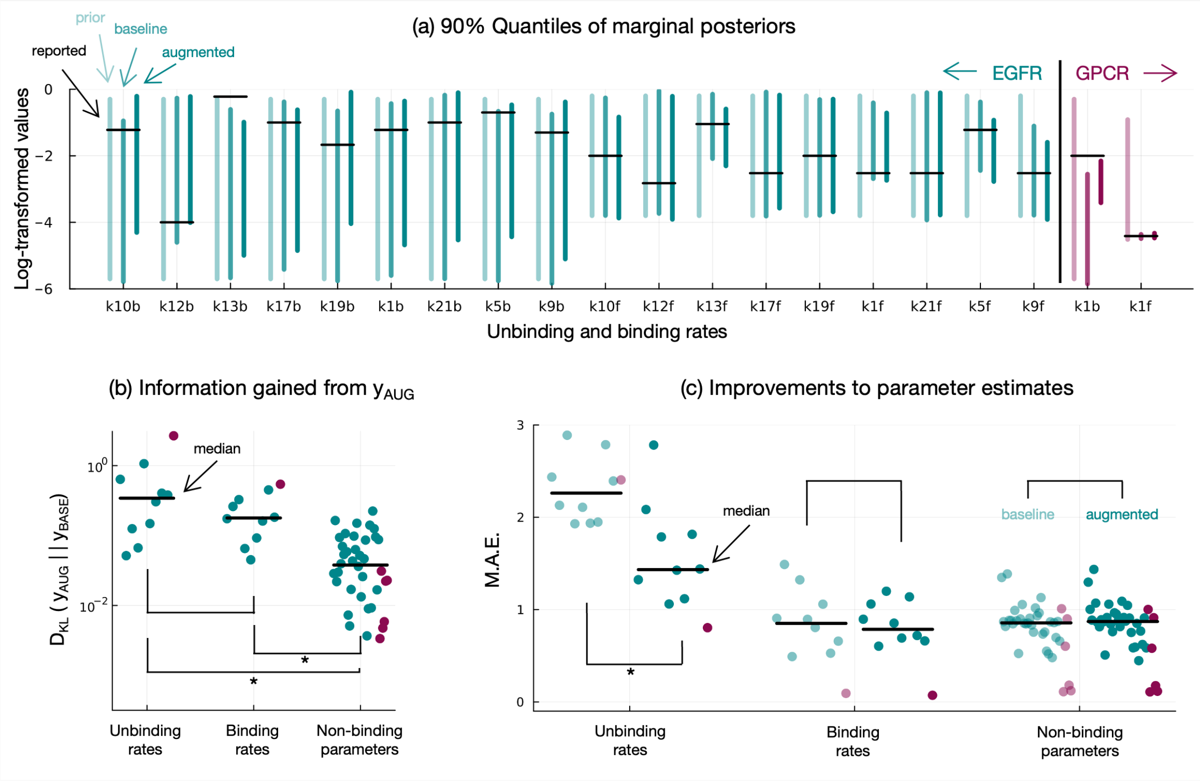

Fig D. Impact of using EGFR protein as test set. Cyan, EGFR results; pink, GPCR results. *p-value < 0.05 (a) 90% quantiles of marginal posterior distributions of binding parameters. All samples on log10 scale. Light cyan line, prior; medium cyan line, baseline posterior; dark cyan line, augmented posterior; black horizontal line, reported parameter value. (b) KL divergence, in bits, from baseline posterior to augmented posterior. Values are grouped by parameter function. (c) Mean absolute error (M.A.E.) of parameter samples. Mean taken with respect to each posterior distribution. Error calculated with respect to the values reported in the literature. Light cyan points, baseline; dark cyan points, augmented; black horizontal line, median M.A.E.

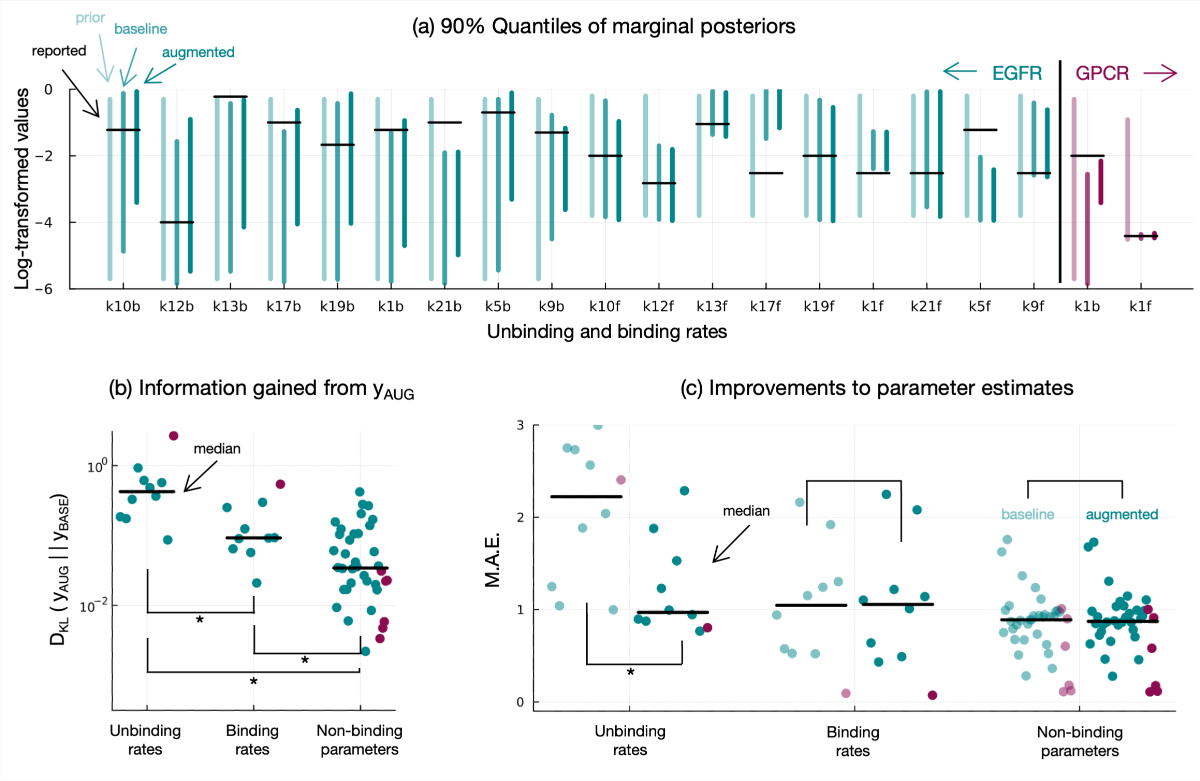

Fig E. Impact of using PLCγ protein as test set. Cyan, EGFR results; pink, GPCR results. *p-value < 0.05 (a) 90% quantiles of marginal posterior distributions of binding parameters. All samples on log10 scale. Light cyan line, prior; medium cyan line, baseline posterior; dark cyan line, augmented posterior; black horizontal line, reported parameter value. (b) KL divergence, in bits, from baseline posterior to augmented posterior. Values are grouped by parameter function. (c) Mean absolute error (M.A.E.) of parameter samples. Mean taken with respect to each posterior distribution. Error calculated with respect to the values reported in the literature. Light cyan points, baseline; dark cyan points, augmented; black horizontal line, median M.A.E.

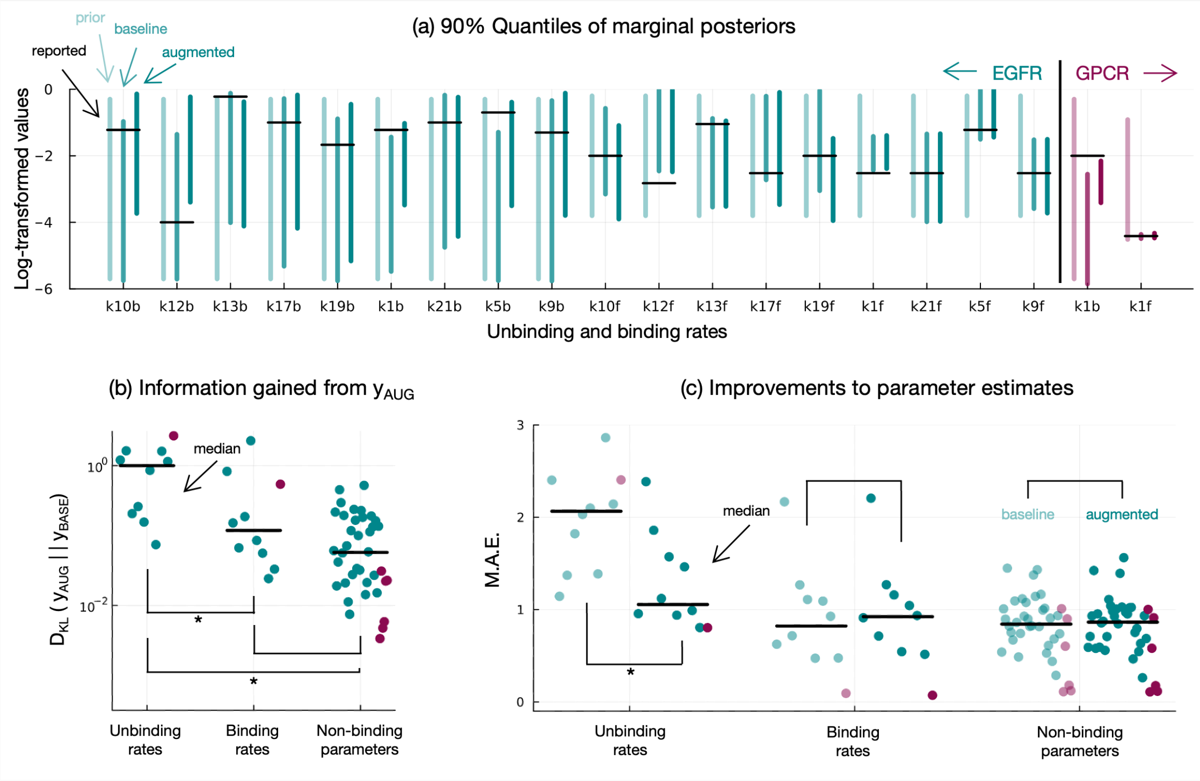

Fig F. Impact of using GRB:EGFR protein as test set. Cyan, EGFR results; pink, GPCR results. *p-value < 0.05 (a) 90% quantiles of marginal posterior distributions of binding parameters. All samples on log10 scale. Light cyan line, prior; medium cyan line, baseline posterior; dark cyan line, augmented posterior; black horizontal line, reported parameter value. (b) KL divergence, in bits, from baseline posterior to augmented posterior. Values are grouped by parameter function. (c) Mean absolute error (M.A.E.) of parameter samples. Mean taken with respect to each posterior distribution. Error calculated with respect to the values reported in the literature. Light cyan points, baseline; dark cyan points, augmented; black horizontal line, median M.A.E.

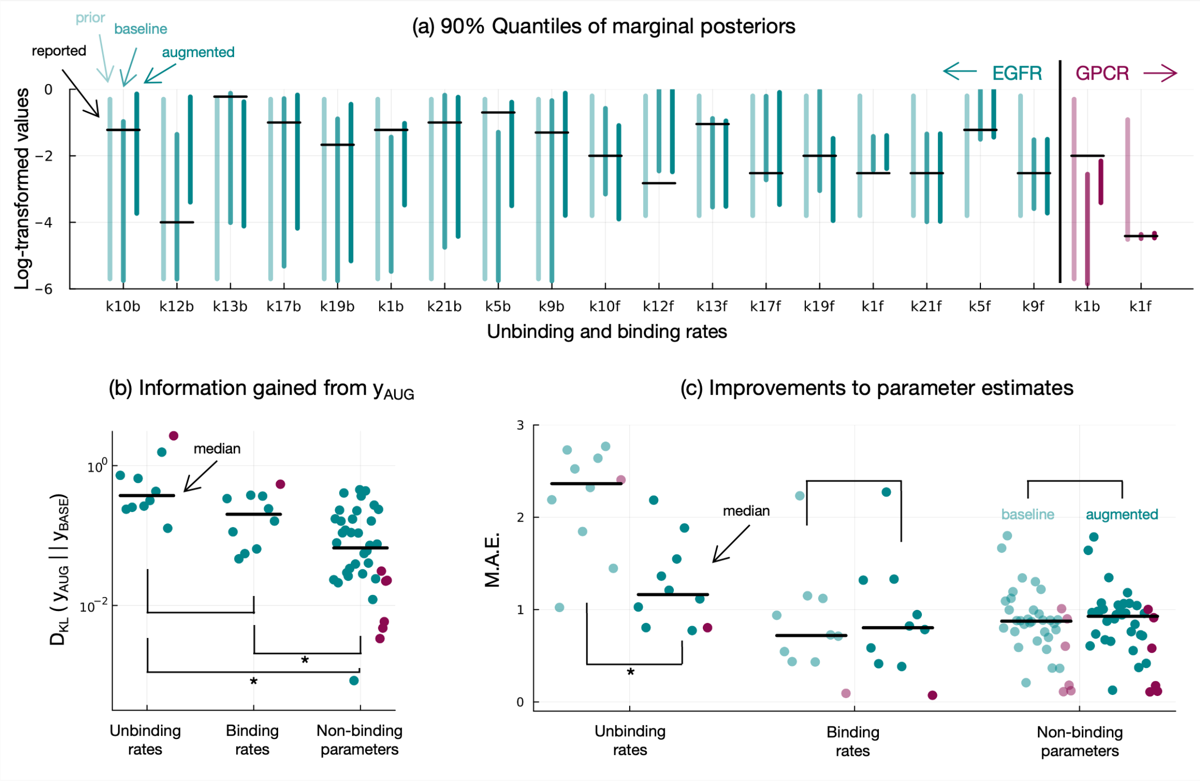

Fig G. Impact of using GRB:SHC2 protein as test set. Cyan, EGFR results; pink, GPCR results. *p-value < 0.05 (a) 90% quantiles of marginal posterior distributions of binding parameters. All samples on log10 scale. Light cyan line, prior; medium cyan line, baseline posterior; dark cyan line, augmented posterior; black horizontal line, reported parameter value. (b) KL divergence, in bits, from baseline posterior to augmented posterior. Values are grouped by parameter function. (c) Mean absolute error (M.A.E.) of parameter samples. Mean taken with respect to each posterior distribution. Error calculated with respect to the values reported in the literature. Light cyan points, baseline; dark cyan points, augmented; black horizontal line, median M.A.E.

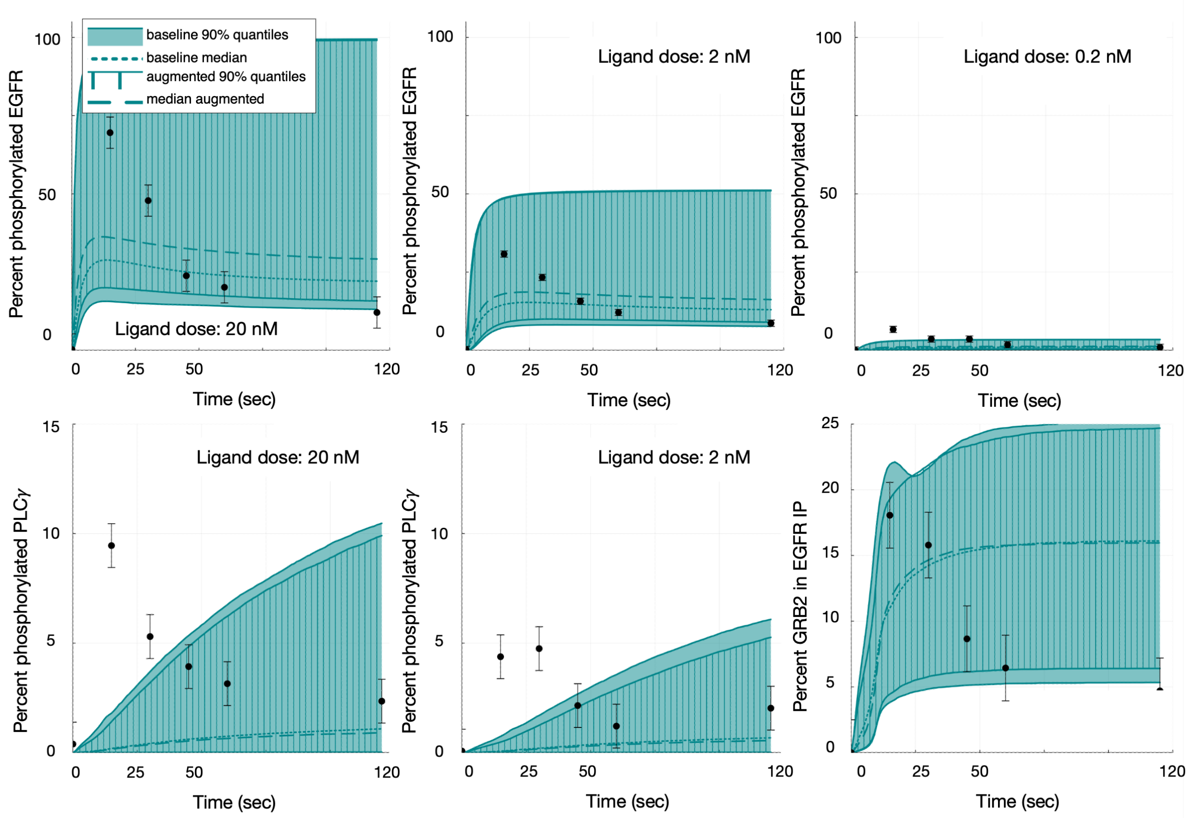

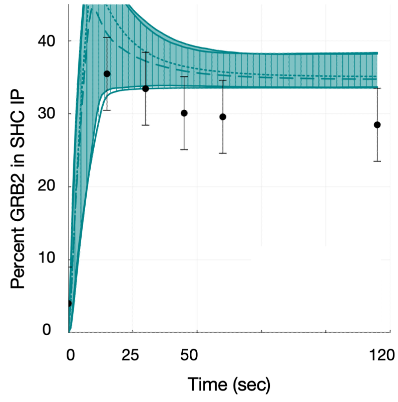

	RMSE		Experimental Error
y_{TEST}	y_{AUG}	y_{BASE}	
EGFR (20 nM)	**17.35**	19.22	5
EGFR (2 nM)	**6.77**	7.63	1
EGFR (0.2 nM)	**2.72**	2.93	1
PLCγ (20 nM)	**4.67**	4.62	1
PLCγ (2 nM)	**2.77**	2.74	1
SHC	60.57	**59.86**	1
GRB:SHC	**24.52**	24.59	5
GRB:EGFR	6.30	**6.19**	2.5

Fig H. Predictions for leave-one-out EGFR test/train split. Cyan, EGFR; pink, GPCR. Shaded region, 90% quantiles of baseline approach; patterned region, 90% quantiles of augmented approach; dotted line, median prediction of augmented approach; dashed line, median prediction of augmented approach; black dots, experimental data with reported error. Root mean square error (RMSE) calculated based on difference between experimental data and median prediction.

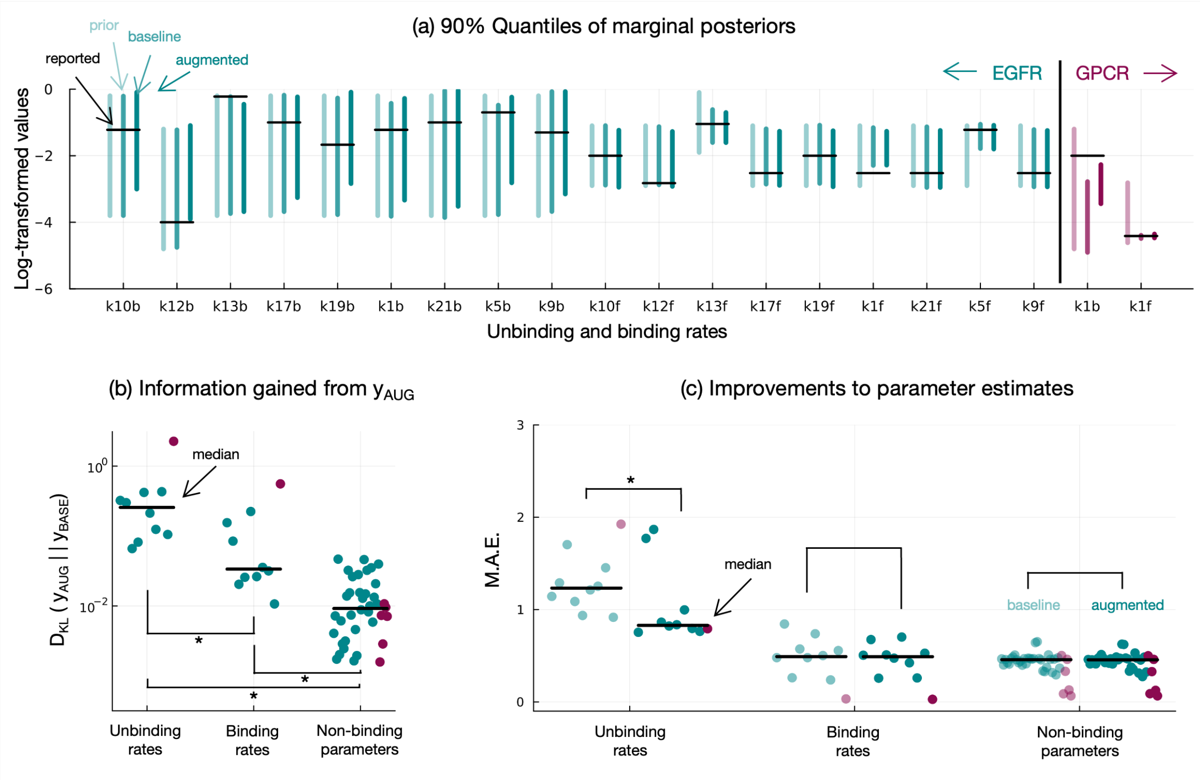

Fig I. Impact of more informative prior on parameter inference. Cyan, EGFR results; pink, GPCR results. *p-value < 0.05 (a) 90% quantiles of marginal posterior distributions of binding parameters. All samples on log10 scale. Light cyan line, prior; medium cyan line, baseline posterior; dark cyan line, augmented posterior; black horizontal line, reported parameter value. (b) KL divergence, in bits, from baseline posterior to augmented posterior. Values are grouped by parameter function. (c) Mean absolute error (M.A.E.) of parameter samples. Mean taken with respect to each posterior distribution. Error calculated with respect to the values reported in the literature. Light cyan points, baseline; dark cyan points, augmented; black horizontal line, median M.A.E.

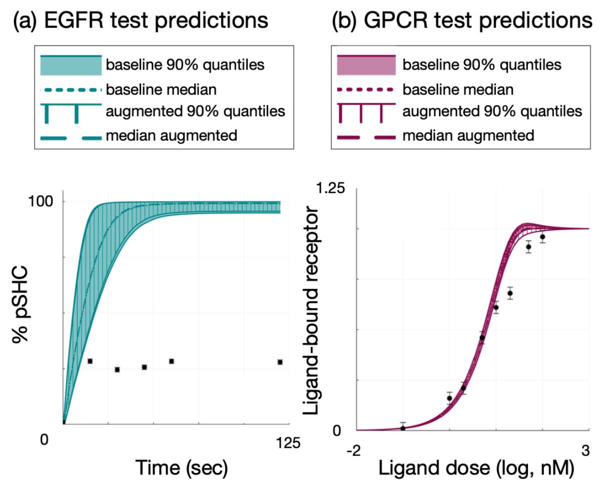

Fig J. Impact of more informative prior on test predictions. (a) Predictions for EGFR test set, the percent of phosphorylated signaling protein SHC from 0-120 seconds. (b) Predictions for GPCR test set, the amount of ligand bound receptor 60 seconds post-stimulation, at different ligand doses, relative to 1000 nM of ligand. Cyan, EGFR; pink, GPCR. Shaded region, 90% quantiles of baseline approach; patterned region, 90% quantiles of augmented approach; dotted line, median prediction of augmented approach; dashed line, median prediction of augmented approach; black dots, experimental data with reported error.

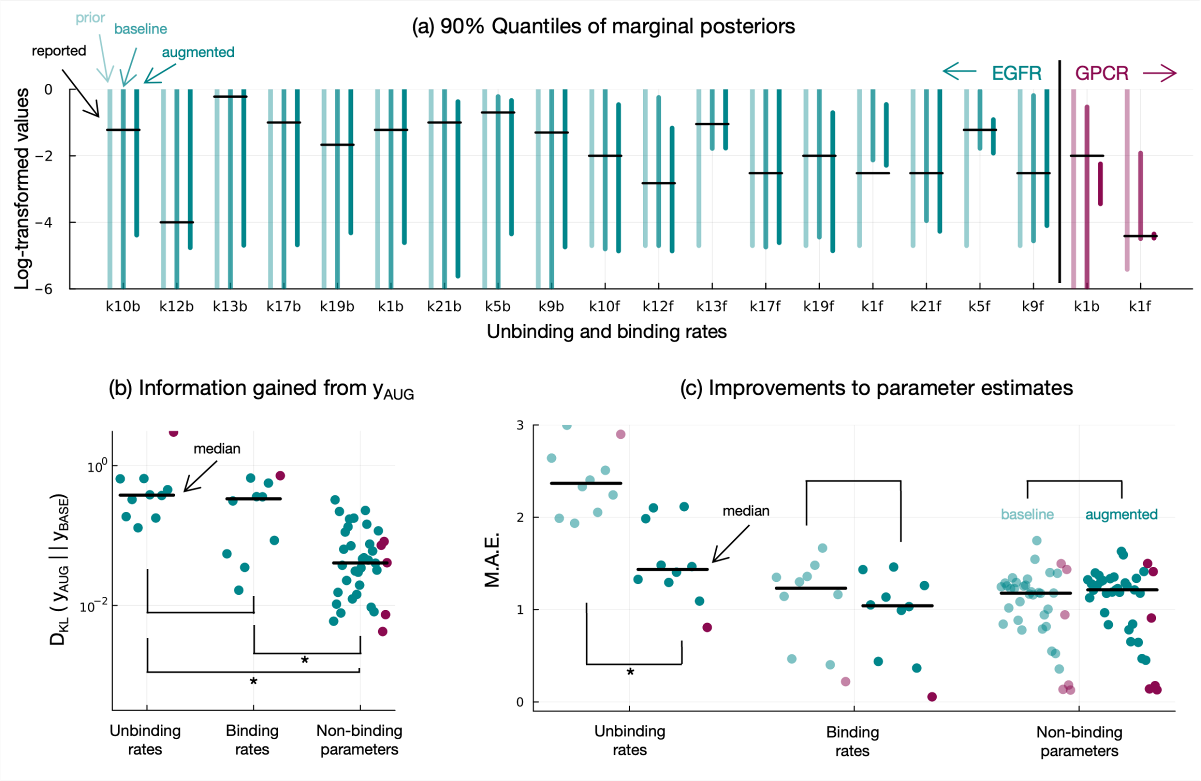

Fig K. Impact of less informative prior on parameter inference. Cyan, EGFR results; pink, GPCR results. *p-value < 0.05 (a) 90% quantiles of marginal posterior distributions of binding parameters. All samples on log10 scale. Light cyan line, prior; medium cyan line, baseline posterior; dark cyan line, augmented posterior; black horizontal line, reported parameter value. (b) KL divergence, in bits, from baseline posterior to augmented posterior. Values are grouped by parameter function. (c) Mean absolute error (M.A.E.) of parameter samples. Mean taken with respect to each posterior distribution. Error calculated with respect to the values reported in the literature. Light cyan points, baseline; dark cyan points, augmented; black horizontal line, median M.A.E.

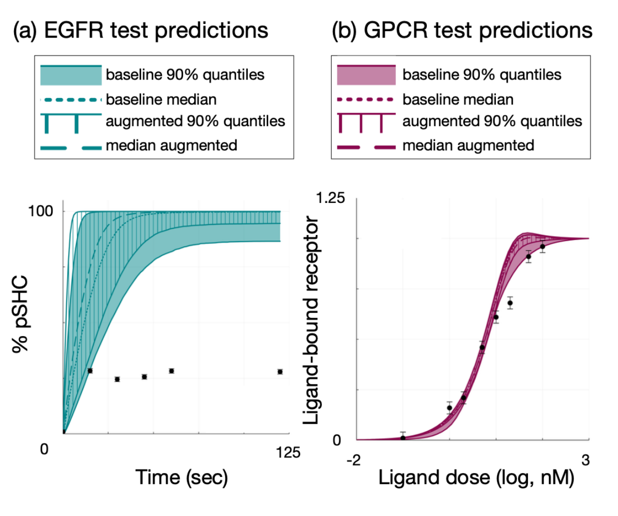

Fig L. Impact of less informative prior on test predictions. (a) Predictions for EGFR test set, the percent of phosphorylated signaling protein SHC from 0-120 seconds. (b) Predictions for GPCR test set, the amount of ligand bound receptor 60 seconds post-stimulation, at different ligand doses, relative to 1000 nM of ligand. Cyan, EGFR; pink, GPCR. Shaded region, 90% quantiles of baseline approach; patterned region, 90% quantiles of augmented approach; dotted line, median prediction of augmented approach; dashed line, median prediction of augmented approach; black dots, experimental data with reported error.

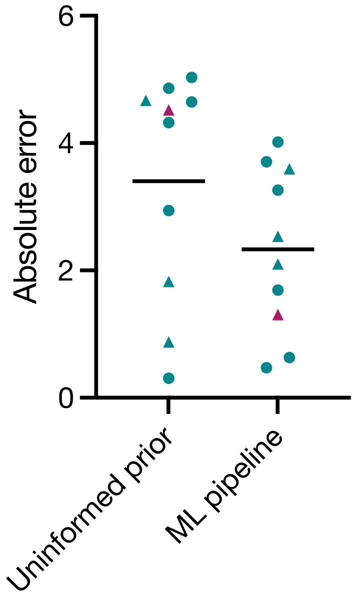

Fig M. ML Pipeline performance using protein complexes with fragments. Cyan, EGFR reaction; pink, GPCR reaction. Circles, K_D predicted using predicted structure; triangles, K_D predicted using experimental structure. (a) Mann-Whitney test comparing absolute error of structure-informed K_D prediction with a random sample from a log-uniform distribution. n=10 binding reactions. *p-value< 0.05. Black horizontal line, sample mean.

3. Appendix References

1. Huijser D, Goodman J, Brewer BJ. Properties of the Affine Invariant Ensemble Sampler in high dimensions [Internet]. arXiv; 2017 [cited 2025 May 16]. Available from: http://arxiv.org/abs/1509.02230

2. Foreman-Mackey D, Hogg DW, Lang D, Goodman J. emcee : The MCMC Hammer. Publ Astron Soc Pac. 2013 Mar;125(925):306–12.

3. Stan Development Team. Stan Modeling Language Users Guide and Reference Manual [Internet]. 2024. Report No.: Version 2.34. Available from: https://mc-stan.org

4. Romero-Molina S, Ruiz-Blanco YB, Mieres-Perez J, Harms M, Münch J, Ehrmann M, et al. PPI-Affinity: A Web Tool for the Prediction and Optimization of Protein–Peptide and Protein–Protein Binding Affinity. J Proteome Res. 2022 Aug 5;21(8):1829–41.

5. Anslyn EV, Dougherty DA. Modern physical organic chemistry. Sausalito, CA: University Science; 2006. 1095 p.
