# Supplementary material for: A multiscale, Bayesian inference approach to augment mechanistic models of cell signaling with machine-learning predictions of binding affinity
Source: PLoS Comput Biol. 2026 Jun 5;22(6):e1014321. doi: 10.1371/journal.pcbi.1014321 (PMC13258154; doi:10.1371/journal.pcbi.1014321)
Supplement: Attachment — Submitted filename: PLCB_Huber.pdf [file pcbi.1014321.s002.pdf]

Report on manuscript PCOMPBIOL-D-25-01377

The manuscript "Multiscale Probabilistic Modeling: A Bayesian Approach to Augment Mechanistic Models of Cell Signaling with Machine-Learning Predictions of Binding Affinity" by Huber et al. showcases a new approach for incorporating structural protein data in inference of binding rates using mechanistic mathematical models. The problem in question is highly relevant, and likely of interest to readers of PLOS Computational Biology. However, I have concerns about the assumptions implicit to the approach, and the presentation of the work, that I feel should be addressed before the paper could be considered suitable for publication.

1. The authors present their work as a "multiscale, probabilistic modelling framework" that leverage a "ML pipeline". This is, for the most part, fine, but it is only in the methods section that how the framework works becomes clear. As far as I can tell, the authors are essentially applying an ML pipeline on sequence/protein data to construct a better prior that can then be used in a Bayesian inference routine. Can the story be presented as such to make it clearer? At least, the introduction could be reworked to make the framework itself clear from the start. Lastly, is there a benefit to using the word "probabilistic" to describe a standard Bayesian inference routine?
2. Only in Section 4.8 (far too late) is it explained that the ML pipeline estimates K_D with $\hat{K}_D$ (the notation should be made clear from the start), and produces both a mean and standard error using SVM regression. I am not yet convinced that one can interpret the resultant distribution constructed as a prior. In particular, how does the SVM approach quantify uncertainty? Earlier in the manuscript, the authors discuss how they "weight" the ML estimate for K_D. How does this interact with the effective prior assumed when incorporating time-series data?
3. The examples presented use the ML pipeline to estimate only K_D. Why can the authors not predict the binding rates themselves? Is it relevant that K_D relates to the steady state ratio of compound to constituent?
4. It is well known that reaction rates vary with, e.g., temperature, pH, etc. However, the ML pipeline doesn't consider such external factors. Can the authors comment on whether this is effectively accounted for in their approach?
5. It would help the story if the authors could be more explicit in their comparison between $\hat{K}_D$ (estimated from the ML pipeline) and K_D estimated directly from the data. The authors currently focus on the improvement in parameter estimate uncertainty, but another important point is validation: are these independent estimates of the same parameter consistent?
6. Why are experimental measurements in Fig 5a completely different from the model predictions? It would also be helpful to add the data points to the legend.
