# Supplementary material for: A multiscale, Bayesian inference approach to augment mechanistic models of cell signaling with machine-learning predictions of binding affinity
Source: PLoS Comput Biol. 2026 Jun 5;22(6):e1014321. doi: 10.1371/journal.pcbi.1014321 (PMC13258154; doi:10.1371/journal.pcbi.1014321)
Supplement: Attachment — Submitted filename: Response to Reviewers_final.docx [file pcbi.1014321.s004.docx]

Response to Reviewers

We thank the reviewers for their constructive reviews. Below, we have provided a response to each comment, written in red font, and indicated how we have edited the manuscript to address the comments. Additionally, we have provided a file that highlights the changes made to the manuscript; the line numbers below refer to that file (“Revised Manuscript with Tracked Changes.docx”).

**Reviewer 1:**

Summary

Huber and Finley present a Bayesian framework for predicting kinetic parameters in mechanistic ODE models, with applications to ERBB and GPCR signaling pathways. While the topic is of clear interest and relevance, the study falls short in several key areas. The results lack sufficient strength to convincingly support the proposed approach. Moreover, the manuscript is difficult to follow due to its diffuse and at times disorganized presentation. A substantial rewrite is necessary to improve readability.

Major Comments
\### Technical Details & Presentation
- **Predictions**: The rationale for choosing SHC in the EGFR model is unconvincing. Given that many model species already have direct clinical relevance, predictions should be done in a leave-one-out fashion. This would also increase the number examples in the test set to something more meaningful.

We thank the reviewer for this feedback and acknowledge that different/test train splits can strengthen the generalizability of our results. To address this feedback, we have added the results of different test/train splits to the supplementary materials (Figures S8 – S12).

- **Baselines**: The evaluation of predictions does not include any positive/negative baselines. How difficult/out-of-distribution are those predictions?

We thank the reviewer for this feedback and acknowledge that more context for the K_D_ values we predict can help the reader contextualize the difficulty of the predictions. PPI-Affinity was benchmarked on several, curated protein-protein and protein-peptide binding datasets. The K_D_’s comprising these benchmarks span several orders of magnitude. The binding affinities we seek to predict fall well within these benchmarks, increasing the likelihood that the EGFR and GPCR systems are within the domain of applicability for PPI-Affinity. To address this feedback, we have added a comparison of the K_D_’s we predict to a representative sample of K_D_ values in the Approach and Results section (Lines 250-254) as well as a discussion of how this test set could be expanded in the Discussion section (Lines 526-533)

\### **Overall Structure and Clarity**
- **Introduction Placement and Focus**: The comparison of this study with existing literature is more appropriate for the _Discussion_ section, where it can be contextualised against the findings. In its current position in the _Introduction_, it disrupts the logical flow.

We thank the reviewer for this feedback. We note that providing background on current approaches and how our method differs from them can be an important part of the introduction. However, we acknowledge that the introduction’s narrative flow can be improved. In the revised manuscript, we have shortened and re-organized the introduction such that the comparison of this study with the literature does not interrupt the explanation of our approach (Lines 53-58).

- **Narrative Coherence**: The introductions frequently shifts between background information and descriptions of the current study, which affects readability.

We thank the reviewer for this feedback and acknowledge that the introduction’s narrative flow can be improved. To address this feedback, we have shortened and re-organized the introduction such that transitions between background information and descriptions of the current study are minimized (Lines 53-58).

- **Conciseness**: Explanations are often overly elaborate and could be significantly streamlined. I would expect that the text can be shortened by at least 25-50%.

We thank the reviewer for this feedback and acknowledge that some explanations can be shortened. In particular, we have made the introduction more concise, as well as the approach and results, where we deleted what was section 2.6. Overall, this paper has been shortened from 7737 words (excluding citations, abstract, and author summary) to 7387 words.

\### **Content and Terminology**
- **Dataset and Model Overview**: The manuscript would benefit from a clearly organised section that outlines the datasets used, the machine learning predictors employed, and the structure of the ODE models. This should be presented early in the methods or a dedicated overview section.

We thank the reviewer for this feedback and acknowledge that a clear overview of these important study features would benefit the reader. To address this feedback, we have added two new sections: “2.1. Cell Signaling ODE Models” and “2.2 Data Augmentation” to the Approach and Results (Lines 195-227).

- **Terminology Use**: Avoid introducing technical terms such as _ybase_ and _yaug_ in the introduction. These are best introduced in the methods or results sections, where they can be explained in the context of their use.

We thank the reviewer for this feedback and have removed these terms from the introduction to section “2.2. Data Augmentation” in the Approach and Results.

- **Figure Descriptions**: Explanations related to figure layout and visual elements should be moved to the figure captions. The main text should focus on interpreting the results, not describing the figure composition.

We thank the reviewer for this feedback and have endeavored to shorten or delete descriptions of the figure composition from the main text. For example, what were previously lines 264-268.

- **Sections 2.5–2.7**: These sections do not add substantive value to the manuscript and could be removed or significantly condensed to maintain focus and improve pacing.

We thank the reviewer for this feedback and acknowledge that the narrative focus of the work can be improved. We have removed section 2.6 but note the importance of sections 2.5 and 2.7:

- 2.5. Investigation of why certain ODE model predictions are changed by our approach compared to others: This provides guidance as to when (or when not) to apply our proposed approach.

- 2.7. Investigation of sensitivity of results to the prior distribution: This is good practice when employing Bayesian inference, given the subjectivity of the prior distribution.

However, we agree that these sections can be improved and have streamlined the text in each of these sections.

**Reviewer 2:**

The manuscript “Multiscale Probabilistic Modeling: A Bayesian Approach to Augment Mechanistic Models of Cell Signaling with Machine-Learning Predictions of Binding Affinity” by Huber et al. showcases a new approach for incorporating structural protein data in inference of binding rates using mechanistic mathematical models. The problem in question is highly relevant, and likely of interest to readers of PLOS Computational Biology. However, I have concerns about the assumptions implicit to the approach, and the presentation of the work, that I feel should be addressed before the paper could be considered suitable for publication.

1. The authors present their work as a “multiscale, probabilistic modelling framework” that leverage a “ML pipeline”. This is, for the most part, fine, but it is only in the methods section that how the framework works becomes clear. As far as I can tell, the authors are essentially applying an ML pipeline on sequence/protein data to construct a better prior that can then be used in a Bayesian inference routine. Can the story be presented as such to make it clearer? At least, the introduction could be reworked to make the framework itself clear from the start. Lastly, is there a benefit to using the word “probabilistic” to describe a standard Bayesian inference routine?

We thank the reviewer for this feedback and acknowledge that the framework can be introduced more clearly; the reviewer is essentially correct in their description of the framework, though we note that the ML pipeline is specifically used to augment the likelihood (rather than the prior distribution). To address this feedback, we have added the description of the framework to the introduction (lines 73-74), shortened the introduction, and added section 2.2, which provides details about the proposed inference framework that complement the introduction. We also remove “probabilistic” in several places and replace it with Bayesian.

2. Only in Section 4.8 (far too late) is it explained that the ML pipeline estimates KD with KD

(the notation should be made clear from the start), and produces both a mean and standard error using SVM regression. I am not yet convinced that one can interpret the resultant distribution constructed as a prior. In particular, how does the SVM approach quantify uncertainty? Earlier in the manuscript, the authors discuss how they “weight” the ML estimate for KD. How does this interact with the effective prior assumed when incorporating time-series data?

We thank the reviewer for this feedback and acknowledge that an earlier introduction of the K_D_ notation would be beneficial—we now introduce it in section 2.2.

The SVM regression model reports the standard error on a dataset of hundreds of independent binding affinity predictions; we acknowledge that more details about the SVM model would be helpful and have added details about the training datasets (lines 250-254)

For parts of the investigation, we previously augment the likelihood term to account for AlphaFold3’s confidence. However, at the request of Reviewer 1, we now exclude these results to streamline the paper’s narrative. We now discuss weighing the ML estimate for K_D_ in the discussion section (lines 470-475), as a possible future direction.

3. The examples presented use the ML pipeline to estimate only KD. Why can the authors not predict the binding rates themselves? Is it relevant that KD relates to the steady state ratio of compound to constituent?

We thank the reviewer for this feedback and acknowledge that, indeed, a direct estimate of the binding rates themselves would be preferable to an estimate of K_D_ when estimating the parameters of a signaling model. However, in contrast to binding affinity, the prediction of kinetic off and on rates has received considerably less attention in the computational chemistry field. Thus, when we evaluated existing models for predicting binding rates, we found them to be less generalizable than K_D_ predictors. In the revised manuscript, we now directly address why we predict KD, rather than the binding rates themselves, in the discussion section (lines 527-532).

We also acknowledge the relationship between the steady state ratio and K_D_; this relationship is another way to define K_D_ compared to the binding off and on rates [1]. The definition of K_D_ in terms of off and on rates is dependent on the binding reaction being bi-molecular; we make sure to emphasize this constraint in lines (239-242).

4. It is well known that reaction rates vary with, e.g., temperature, pH, etc. However, the ML pipeline doesn’t consider such external factors. Can the authors comment on whether this is effectively accounted for in their approach?

We thank the reviewer for this feedback and acknowledge that we should clarify how environmental factors are accounted for in our approach. As in other ML pipelines, our approach accounts for temperature when calculating the reaction rates [2]. We state this in section 4.5 (lines 648-649).

5. It would help the story if the authors could be more explicit in their comparison between KD (estimated from the ML pipeline) and KD estimated directly from the data. The authors currently focus on the improvement in parameter estimate uncertainty, but another important point is validation: are these independent estimates of the same parameter consistent?

Here, the reviewer We thank the reviewer for this feedback suggesting we highlight the validation of our framework via comparing KD estimated with the ML pipeline to KD estimated directly from the data. We agree that the accuracy of the predictions is equally important to the changes in uncertainty. We now implement an additional paired comparison of these parameters, per Reviewer 4’s recommendation (Line 327).

6. Why are experimental measurements in Fig 5a completely different from the model predictions? It

would also be helpful to add the data points to the legend.

We thank the reviewer for raising this point. Interestingly, the difference exists for both parameter estimation approaches and in the predictions of the original publication by Kholodenko and coworkers. This indicates that the difference between model predictions and experimental measurements is not caused by the difference in parameter estimation approaches. Our present work focuses on presenting the parameter estimation pipeline, and we have not included an in-depth study of the difference between data and model simulations, which may be due to the structure of the model itself (i.e., equations), rather than model parameters. However, we agree with the reviewer that it is important to acknowledge the difference. To better clarify this point, we now establish that this difference is present in the original publication earlier in the paper (lines 214-220; the figure with data from the original papers is now Fig. 2, rather than Fig. 7).

**Reviewer 3:**

Reviewer #3: This work proposes to use available databases to improve parameter inference of models of cell signaling. In particular, they aim to augment sparse time-series data of concentrations (typically used for inference) with measurements of protein and amino acid sequence structure that have become available. This data is used in a ML pipeline that predicts binding affinity parameters of cell signaling models. Using a Bayesian inference approach and the KL divergence metric between distributions, they quantify the information gained from incorporating these additional sequence datasets. They apply their approach to EGFR and GPCR signaling models. The use of Alpha Fold and other ML models to bridge the scale of sequence/structure to the scale of protein concentrations was interesting and new to me. There is also the potential to generalize the approach to other models. Overall, the paper is well-written so I recommend minor revisions (that mostly have to do with incorporating background and methods information better into results) as follows:

1. On page 10, where the authors mention that “One of these _D predictions was made with an experimental structure”, it would be good to already clarify that the experimental structure was available for GPCR signaling, and that all others are based on AlphaFold predicted structures.

We thank the reviewer for this feedback and have introduced an overview of the sequence and structure datasets used in the paper in the new section 2.2 “Data Augmentation” (Table 2). This overview indicates how the structural data belongs to the GPCR system and sequence data belongs to the EGFR system.

2. I think the manuscript would be improved if the authors mentioned earlier that their choice of the two test cases shows scenarios that vary in model complexity, in the number of unknown parameters, in the availability of experimental data, etc.

We thank the reviewer for this feedback and have added this comparison in the first section of the results, section 2.1, lines (196-203).

3. Section 2.3 begins quite abruptly with results of Bayesian inference, but not much is known at this point about the nature of the ODE models, the type of data used for parameter estimation, the data used for testing, etc. This is all addressed in Materials and Methods, but it feels like some of this background is needed here to properly understand the setting of the pipeline and results.

We thank the reviewer for this feedback and collectively respond to these three points together, as we now introduce the ODE models and datasets in the first and second section of the results (newly added for the revised manuscript: section 2.1 and 2.2).

4. In figure 4c), is there any reason why the baseline distribution for non-binding parameters is tighter than for the other parameter categories?

We limit the bounds for the non-binding parameters to be 2 orders of magnitude above and below the reported value. For other parameters, we align the bounds distributions found in prior work. We clarify this in the revised manuscript (lines 694-695).

5. In the paragraph starting on line 251, the authors discuss that output other than the “test set” may look qualitatively different in predictions from their baseline vs augmented parameter estimation. As mentioned earlier, it is not clear at this point what this means. While this is clarified later, I think it is important to mention it here to be able to distinguish between the different data predictions.

We thank the reviewer for this feedback and now introduce the test/train datasets in the second section of the results (section 2.2).

6. Figure 6 lists panel d) before panel c).

This has been corrected!

7. Typo on line 433, I think it should say “are more stringent than the default…”

The reviewer is correct; this has been updated.

8. In Materials and Methods, the authors say that “To calculate the projected change in an output due to a 5% change in a particular parameter, we multiple the local sensitivity by 5% of the maximum likelihood parameters.” I haven’t seen this approached in this way before. Is there a rigorous basis for doing this?

We based our approach on the local sensitivity analyses laid out in *Mathematical Modeling In Systems Biology* by Brian Ingalls. We aim to perform a local sensitivity analysis to provide the derivative in an ODE model species with respect to a particular parameter given a small perturbation (here, a 5% change) in the parameter values through a linearization formula. We agree with the reviewer that we have not provided a justification of the selection of 5% and have added a citation to Ingalls in the explanation of the local perturbation analysis (Lines 751).

**Reviewer 4**

Reviewer #4: Summary: This study introduces a multiscale probabilistic framework that integrates machine learning-derived binding affinity estimates into mechanistic models of cell signaling. The authors use protein structure data from PDB and AlphaFold, along with sequence information from UniProt, to infer kinetic parameters, such as binding and unbinding rates, for receptor systems including EGFR and GPCRs. A data augmentation strategy incorporates predictions from protein structure models into the Bayesian inference process. Changes in the posterior distributions of binding parameters, including shifts in uncertainty and central estimates, are quantified using Kullback-Leibler divergence. These inferred parameters are then used to simulate downstream signaling dynamics, which are compared to experimental measurements to assess predictive consistency.

Strengths and innovation
1. Multiscale data integration: The framework bridges structural and systems biology by combining molecular-scale information, e.g., protein sequences and AlphaFold-predicted structures, with dynamic models of cell signaling.
2. Structure-informed parameter inference: The study incorporates binding affinity estimates derived from protein structure models, both experimental and predicted, into the inference of kinetic parameters, enabling application even in the absence of crystal structures.
3. Bayesian framework with Kullback-Leibler (KL)-based evaluation: A Bayesian approach is used to infer binding and unbinding rates, and changes in posterior distributions due to data augmentation are quantified using KL divergence.
4. Application to receptor signaling pathways: The method is demonstrated on EGFR and GPCR systems, showing a pipeline for integrating structural predictions into dynamic pathway modeling.

We thank the reviewer for this helpful feedback on the strengths of our work!

Areas of improvement

The approach proposed by the authors is highly creative: it leverages crystal structures, or AlphaFold-predicted structures when experimental data is unavailable, to estimate binding affinities, which are then used to inform parameter inference in signaling models. While this multiscale strategy is compelling, it also presents important challenges.

Inferring binding affinities from AlphaFold-derived structures is very challenging. AlphaFold and related structural models are designed to predict a single, static apo conformation, which may not correspond to the binding-competent state required for accurate kinetic parameter inference. This limitation is particularly pronounced for flexible proteins such as EGFR and GPCRs, where conformational heterogeneity is known to be essential for binding. Using only the lowest-energy apo structure may lead to inaccurate or biased estimates. To evaluate the robustness of their approach, the authors could apply their pipeline to both apo and holo structures from the PDB for EGFR and representative GPCRs. Comparing predicted KD values across conformational states would help determine the sensitivity of their predictions to structural flexibility and state-specific effects.

An even better test would involve identifying or generating molecular dynamics (MD) simulations of EGFR and representative GPCRs. Such simulations provide ensembles of conformations sampled across biologically relevant timescales, capturing the dynamic nature of these flexible proteins. The authors could apply their KD prediction pipeline to different conformations from the MD ensembles and quantify the variance in predicted KD values, hence, assessing whether the model meaningfully captures the impact of conformational heterogeneity on binding affinity, or whether relying on static structures systematically biases the predictions.

We thank the reviewer for this feedback and agree that protein conformation is a vital consideration in fully elucidating the relationship between structure and function. Indeed, protein structure is dynamic and presents an exciting area of development for structural-derived models like AlphaFold. In this work, we establish that protein sequence or structure data can be used to improve cell signaling models, using ML models like AlphaFold as-is. We would propose that this finding is valuable because of the direct application of such ML models. We also recognize that such direct application should be done with caution; to demonstrate this, we included a section investigating the impact of ML confidence on framework performance (Figure 4b). However, we agree that the reviewer’s suggestion to investigate the impact of conformation on the proposed approach is a valuable suggestion. With this in mind, we have addressed the reviewer’s feedback by discussing an in-depth investigation on the effect of conformation change on binding affinity prediction performance as an intuitive next step in refining our proposed approach (Lines 534-540).

Figure 3: The figure suggests that the structure-informed ML pipeline improves prediction accuracy of binding affinity (KD) relative to a log-uniform prior. However, the sample size is small (n = 10 binding reactions), which constrains the statistical power of the Mann-Whitney U test (was the p-value computed using an exact test or an asymptotic approximation?). Second, the assumption of independence may not hold, particularly in the ML pipeline group, where multiple predictions per reaction (e.g., from different AlphaFold-generated structures) may have been included. Treating these as independent observations could artificially inflate statistical significance. Additionally, the high variance in prediction error, for both the uninformed baseline and the ML pipeline, raises concerns about the practical utility of the results. For instance, although the mean error for the ML pipeline is lower, the large standard deviation suggests that worse predictions remain reasonably likely. Overall, while the figure suggests improvement, stronger statistical validation and clearer definitions of replicates are needed.

We thank the reviewer for this feedback and acknowledge that clearer definitions of replications with respect to Figure 3 would be beneficial. Given the significant relationship between AF3 model confidence and KD prediction error, we used only the highest-confidence AF3 prediction per binding reaction; this was not previously addressed in the text, so we added this clarification in the revised manuscript (lines 652-654). Given the small sample size, the Mann-Whitney U test was computed with an exact test. This provides more strength to the statistical validation. To address the reviewer’s point, this methodological facet has been added to clarify the strength of the statistical validation (Lines 761-762). Finally, we acknowledge that the improvements shown in Figure 3 are not absolute. As the reviewer notes, the standard deviation suggests worse predictions are still reasonably likely. Still, there is a statistically significant improvement in mean absolute error. To address this caveat to our results, we have added a more in-depth discussion of the results of Figure 3 (Lines 454-456).

Figure 4 evaluates the impact of data augmentation on parameter inference. Panel (a) shows 90% quantiles of marginal posterior distributions of binding parameters, indicating that augmented data generally narrows posterior distributions. While this suggests increased certainty, the direction of shift is not uniformly beneficial: for some parameters (e.g., k12b, k1f, k1b), the augmented posterior appears to move away from the ground truth. Could the authors apply a statistical test, such as a paired comparison of MAE across conditions or Bayesian model selection, to assess whether the augmented posteriors yield more accurate estimates, not just tighter ones? Panel (b) quantifies information gain using KL divergence, which reflects changes in distribution but not accuracy. While it confirms that data augmentation alters the posteriors compared to the baseline, it does not indicate whether the augmented estimates are closer to the true values. Panel (c) is more informative, showing that augmentation shifts the posterior distributions of unbinding parameters and reduces the mean absolute error (MAE), while binding and non-binding parameters remain largely unchanged. The lack of effect on non-binding parameters is expected, as the augmented data specifically targets binding interactions. However, the limited impact on binding parameters is less intuitive, given their coupling with unbinding rates. Could the authors clarify why augmentation selectively improves unbinding estimates? Is this due to the structure of the likelihood function, the nature of the ML predictions (e.g., affinity being more sensitive to unbinding), or another modeling constraint?

We thank the reviewer for this feedback and acknowledge that an assessment of whether posteriors yield more accurate estimates, not just tighter ones, is crucial for the work. To assess gains in accuracy, we employed paired t-tests of the MAE with respect to the posterior distribution (Figure 4c). To make it clearer that a paired comparison of MAE was conducted, we have added Lines 327 to the results section. We also acknowledge the interesting result that augmentation selectively improves unbinding rates, irrespective of whether we change the prior distribution or test train split (Lines 490-492).

Figure 5 shows that data augmentation has only a moderate effect on the baseline model’s predictive performance. In the EGFR panel, neither model captures the experimental data well, particularly SHC phosphorylation dynamics, raising concerns about the adequacy of the model or its parameterization. The authors should clarify whether this mismatch stems from model limitations, training data quality, or the scope of the augmented information. In the GPCR panel, both models better match experimental data, but the differences are minimal (or at least, not observable in the figure). It remains unclear whether augmentation improves model fit. A quantitative comparison, e.g., RMSE, likelihood scores, or posterior predictive checks, would help determine whether the improvement is statistically significant.

We thank the reviewer for this feedback and acknowledge the merits of a quantitative comparison. To address this point, we have added panel C to Figure 6 (Fig. 5 in the original manuscript), which provides a quantitative comparison of the goodness of ODE model predictions with and without data augmentation. We acknowledge that this clear difference between model simulations and experimental data is important to explain. Interestingly, the difference exists for both parameter estimation approaches and in the predictions of the original publication. Our present work focuses on presenting the parameter estimation pipeline, and we have not included an in-depth study of the difference between data and model simulations, which may be due to the structure of the model itself (i.e., equations), rather than model parameters. However, we agree with the reviewer that it is important to acknowledge the difference. To better clarify this point, we now establish that this difference is present in the original publication earlier in the paper (lines 214-220; the figure with data from the original papers is now Fig. 2, rather than Fig. 7)

Small comments:
The authors should provide a clear mathematical description of the signaling network model used for parameter inference, including the system of differential equations, model parameters, and underlying kinetic assumptions. This would clarify how structural priors influence dynamic outputs. Additionally, more detail on the Bayesian framework and the SVM-based PPI-Affinity model would improve transparency, specifically, information on the feature space, kernel type, and training data, which are essential for assessing prediction reliability and reproducibility.

We thank the reviewer for this feedback and have added the ODE system reaction diagram, system model parameters, and underlying kinetic assumptions to section 2.1. Similarly, we have added more details about the PPI-Affinity model to the methods and results including information on the kernel type (647), and training data (250-254). Finally, we have presented details of the Bayesian framework earlier in the work (section 2.2).

**References**

[1]	T. D. Pollard, “A Guide to Simple and Informative Binding Assays,” *Mol. Biol. Cell*, vol. 21, no. 23, pp. 4061–4067, Dec. 2010, doi: 10.1091/mbc.e10-08-0683.

[2]	L. C. Xue, J. P. Rodrigues, P. L. Kastritis, A. M. Bonvin, and A. Vangone, “PRODIGY: a web server for predicting the binding affinity of protein–protein complexes,” *Bioinformatics*, vol. 32, no. 23, pp. 3676–3678, Dec. 2016, doi: 10.1093/bioinformatics/btw514.
