# Supplementary material for: A multiscale, Bayesian inference approach to augment mechanistic models of cell signaling with machine-learning predictions of binding affinity
Source: PLoS Comput Biol. 2026 Jun 5;22(6):e1014321. doi: 10.1371/journal.pcbi.1014321 (PMC13258154; doi:10.1371/journal.pcbi.1014321)
Supplement: Attachment — Submitted filename: Response to Reviewers_R2.docx [file pcbi.1014321.s005.docx]

**Response to Reviewers**

We thank the reviewers for their constructive reviews. Below, we have provided a response to each comment, written in red font, and indicated how we have edited the manuscript to address the comments. Additionally, we have provided a file that highlights the changes made to the manuscript; the line numbers below refer to that file (“Revised Manuscript-R2 with Tracked Changes.docx”).

**Reviewer #1**

I thank the authors for substantially revising the manuscript; the text now reads much more smoothly and has markedly improved flow. While I find the overarching idea of the manuscript strong, the current results leave me with the impression that, at least in its present form, the combined approach does not yet demonstrate a clear improvement in predictive performance.

We are grateful to Reviewer 1 for recognizing our efforts! The thoughtful reviewer feedback has been helpful in improving the overall work.

\## Major concerns
\### ML pipeline
It is important to clarify (and ideally explicitly verify) that the PPI affinity model was not trained on the same K_D parameters used in this study. Given the stated publication date (2022), it seems plausible that AlphaFold3-predicted structures were not available, but I cannot follow the authors’ claim that no PDB structures were available for any of the complexes in the EGFR model. A quick search yields several relevant structures, for example:

- EGF:EGFR – [https://www.rcsb.org/structure/1IVO](https://urldefense.com/v3/__https:/www.rcsb.org/structure/1IVO__;!!LIr3w8kk_Xxm!sn8l-Y2TG_OFLVAqUlPtMmIbJneMwvu6S_Uli1xrvC_E3FUZzaalpkHaEBsTkRJfM5VVMsU0xlbmeJY$), [https://www.rcsb.org/structure/8HGS](https://urldefense.com/v3/__https:/www.rcsb.org/structure/8HGS__;!!LIr3w8kk_Xxm!sn8l-Y2TG_OFLVAqUlPtMmIbJneMwvu6S_Uli1xrvC_E3FUZzaalpkHaEBsTkRJfM5VVMsU0DziV-ms$)
- SHC1:EGFR (L858R) – [https://www.rcsb.org/structure/5CZI](https://urldefense.com/v3/__https:/www.rcsb.org/structure/5CZI__;!!LIr3w8kk_Xxm!sn8l-Y2TG_OFLVAqUlPtMmIbJneMwvu6S_Uli1xrvC_E3FUZzaalpkHaEBsTkRJfM5VVMsU0Qb2or-s$)
- GRB2:SOS1 (peptides) – [https://www.rcsb.org/structure/1GBR](https://urldefense.com/v3/__https:/www.rcsb.org/structure/1GBR__;!!LIr3w8kk_Xxm!sn8l-Y2TG_OFLVAqUlPtMmIbJneMwvu6S_Uli1xrvC_E3FUZzaalpkHaEBsTkRJfM5VVMsU0khMFd84$)

We thank the reviewer for this feedback and acknowledge that clarity regarding any overlap between our K_D_ predictions and the PPI affinity model training set is important. Explicitly clarifying that the PPI affinity model was not trained on the same K_D_ parameters used in this study would help contextualize the challenge of our inference problem. We have now done so in lines 166-167.

We also acknowledge additional clarity regarding our claim that there were no PDB complexes for the EGFR model would be helpful. We have added a section to the methods detailing out structure selection process (lines 476-487) that explains how we down-selected sequence and structure data, as well as new results (Supplementary Figure S13) that support the assumptions we made when selecting structural data to use here.

Moreover, PPI affinity appears to be intended primarily for protein–peptide binding prediction, whereas the Materials and Methods suggest that full-length sequences of both interactors were used. Together with the fact that many of these interactions are weak, transient, and often mediated by intrinsically disordered regions (precisely the types of interactions where AlphaFold-derived approaches are known to struggle), this warrants a substantially more careful validation of the ML pipeline and its applicability to the present setting.

We thank the reviewer for this feedback and acknowledge that understanding the applicability of our ML pipeline to cell signaling parameter estimation is fundamental to our work. We note that PPI affinity offers two prediction cases: protein-peptide binding prediction and protein-protein binding prediction. We use both in this work, depending on the proper use case. To make this clearer, we have added an additional explanation of the two PPI affinity prediction models in lines 169-171.This explanation makes clearer the applicability of our ML pipeline to the present setting.

We also acknowledge the challenging nature of the interactions we seek to predict. Indeed, Reviewer 4 also brings up this important element of our prediction task. It would be beneficial to add a forward-looking acknowledgement of the challenges inherent with this prediction task. To better acknowledge these challenges, we have thus added a more thoughtful discussion of the elements inherent to binding affinity prediction in the context of signaling proteins (lines 399-407).

This discussion makes it clear that, now that this work establishes the use case of parameter augmentation for cell-signaling models using binding affinity prediction, an in-depth exploration of only the binding affinity prediction for these types of proteins would be an excellent next step. This next step could be embarked upon in close collaboration with computational structural biologists to identify the best test cases that address the limitations that we know AlphaFold faces. Due to the importance of this part of the discussion, we also moved it earlier in the discussion section.

\### Combined pipeline
From Figures 5 and 6, it appears that although parameter estimates improve, the model predictions themselves remain essentially unchanged. This is somewhat disappointing, as it would suggest that the combined mechanistic + ML approach yields limited practical gain beyond parameter regularisation.

One possibility is that the inferred K_D values are non-identifiable, and that the augmentation primarily constrains the ratio of binding and unbinding rates to match the predicted K_D, without altering predictive behavior. This could be tested directly by comparing the baseline, augmented, and ML predicted K_D distributions.

We thank the reviewer for this feedback and acknowledge that the model predictions for some of the experimental outputs remain unchanged. We also acknowledge the reviewer’s concerns about identifiability of K_D_ values. Indeed, when we first noticed the lack of change in the validation set, this led us to question whether the significant improvement in parameter values leads to a noteworthy change in any of the predictions; more broadly, it was vital to provide an analysis that could demonstrate when data augmentation is useful.

To answer this question, we leveraged a sensitivity analysis, which indicates that changes in predictions are driven be sensitivity to unbinding rates (Figure 7; described in lines 288-293). Taken in concert with the respectably constrained parameter quantiles in Figure 5a, this result verified the practical identifiability of the forward and reverse binding rates [1]. This result also provides an important caveat to the impact of our approach: it is most impactful in the case that an output of interest is sensitive to unbinding rates. This has been explicitly stated in lines 385-387.

\### Minor concerns
- Lines 254, 286, 289: “Error! Reference source not found.”

The reference has now been fixed, thank you!

**Reviewer #2**

The authors have addressed the majority of my original concerns. The introduction, in particular, is now much more clear.

We thank the reviewer for acknowledging our improvements!

I am still unconvinced that the ML approach augments the likelihood, although I accept that it probably doesn't matter. Specifically, the output of the ML approach is a predicted combination of the model parameters. I would see this more as adjusting the prior, which is then used with an explicit likelihood to formulate the posterior.

Perhaps the authors could explicitly show how they (mathematically) augment the likelihood with the output of the ML pipeline (e..g., to backup lines 350-352 and Section 4.6). Aon line 521 the authors state that the full details are included in the supplementary information, however the text provided in the SI is not explicit (and again makes it look like a prior placed on K_D).

We thank the reviewer for this feedback and acknowledge that an explicit breakdown of our formulation would be beneficial. We also appreciate the author’s point about the nuance between augmenting prior and likelihood. To address this, we have implemented the reviewer’s suggested changes. Specifically, we have added Table S1 to the Supplement. Table S1 details all prior and likelihood distributions, thereby making the formulation more explicit.

A final minor comment is that it is unclear to me what the authors mean on line 60 when they say that "data on protein sequence is on a different scale". Compared to what? Scale as in the number of data points/reads? Or the measurement scale of the quantities they are interested in?

We thank the reviewer for this feedback and acknowledge that the wording of this sentence could be improved. We intended to compare the measurement scale of the quantities we are interested in for this work. To make this clearer, we have re-worded this sentence (lines 60-61).

**Reviewer #4**

The revision is substantially improved, and the authors’ responses to the reviewers are clear and generally satisfactory. I recommend acceptance.

As a minor, forward-looking note, I encourage the authors to briefly acknowledge in the Discussion the limitations of inferring K_D from AlphaFold-derived structures, particularly in contexts where binding involves conformational selection and/or induced-fit mechanisms upon complex formation. However, I recognize that a deeper analysis may be beyond the scope of the current manuscript, and hence, my overall recommendation is acceptance.

We thank the reviewer for acknowledging our improvements!

We also thank the reviewer for their feedback regarding addressing the limitations of inferring K_D_ from AlphaFold-derived structures. We have implemented the suggested feedback and added a more thoughtful discussion of this task (lines 399-407), which also addresses a similar point raised by Reviewer 1.

**Response References**

[1]	N. J. Linden, B. Kramer, and P. Rangamani, “Bayesian parameter estimation for dynamical models in systems biology,” *PLOS Comput. Biol.*, vol. 18, no. 10, p. e1010651, Oct. 2022, doi: 10.1371/journal.pcbi.1010651.
