# Supplementary material for: A multiscale, Bayesian inference approach to augment mechanistic models of cell signaling with machine-learning predictions of binding affinity
Source: PLoS Comput Biol. 2026 Jun 5;22(6):e1014321. doi: 10.1371/journal.pcbi.1014321 (PMC13258154; doi:10.1371/journal.pcbi.1014321)
Supplement: Attachment — Submitted filename: Response to Reviewers_R3.docx [file pcbi.1014321.s006.docx]

**Response to Reviewers**

We thank the reviewers for their constructive reviews. Below, we have provided a response to each comment, written in red font, and indicated how we have edited the manuscript to address the comments. Additionally, we have provided a file that highlights the changes made to the manuscript; the line numbers below refer to that file (“Revised Manuscript-R3 with Tracked Changes.docx”).

**Reviewer #1**

The authors have reasonably addressed most of my concerns.

We are grateful to Reviewer 1 for their feedback, which has helped improve our work.

**Reviewer #2**

I thank the authors for addressing the majority of my remaining comments. I only have one minor point below that could be clarified before publication.

I can see now that the authors construct a likelihood for K_D using a log normal distribution along with the ML predicted estimate (through f(theta)) and the ML predicted error (sigma). It would be helpful if the authors could be a little more explicit about their assumptions and the transformation from an error estimate to the standard deviation of exp(K_D). Are the estimates (error and point) from the ML approach on the log scale already? Or do the authors argue through the order-of-magnitude change that they are already on the log scale? Particular as, at first glance, the mean and standard deviation are not given by f(theta) and sigma, respectively, but rather transformations thereof.

We thank the reviewer for this feedback and acknowledge that using a log normal distribution, rather than normal distribution, requires us to transform the parameters and data of the distribution such that it is on a log-scale. These transformations were not clear in the notation we provided in Supplementary Information Table S1. To make the transformations clearer, we have updated the table to include all log transformations. We now also refer to this table and section 1.3 of the supplement (Lines 520-521 and 539), which expands upon our transformation for the reported ML pipeline error.
