# Supplementary material for: A multiscale, Bayesian inference approach to augment mechanistic models of cell signaling with machine-learning predictions of binding affinity
Source: PLoS Comput Biol. 2026 Jun 5;22(6):e1014321. doi: 10.1371/journal.pcbi.1014321 (PMC13258154; doi:10.1371/journal.pcbi.1014321)
Supplement: Peer review history

# Peer review: Decision Letter 0

**DOI:** 10.1371/journal.pcbi.1014321.r001

**Authors:** Fabian Spill

2 Sep 2025

PCOMPBIOL-D-25-01377

Multiscale Probabilistic Modeling: A Bayesian Approach to Augment Mechanistic Models of Cell Signaling with Machine-Learning Predictions of Binding Affinity

PLOS Computational Biology

Dear Dr. Finley,

Thank you for submitting your manuscript to PLOS Computational Biology. After careful consideration, we feel that it has merit but does not fully meet PLOS Computational Biology's publication criteria as it currently stands. Therefore, we invite you to submit a revised version of the manuscript that addresses the points raised during the review process.

Please submit your revised manuscript within 60 days Nov 02 2025 11:59PM. If you will need more time than this to complete your revisions, please reply to this message or contact the journal office at ploscompbiol@plos.org. When you're ready to submit your revision, log on to https://www.editorialmanager.com/pcompbiol/ and select the 'Submissions Needing Revision' folder to locate your manuscript file.

Please include the following items when submitting your revised manuscript:

* A rebuttal letter that responds to each point raised by the editor and reviewer(s). You should upload this letter as a separate file labeled 'Response to Reviewers'. This file does not need to include responses to formatting updates and technical items listed in the 'Journal Requirements' section below.

* A marked-up copy of your manuscript that highlights changes made to the original version. You should upload this as a separate file labeled 'Revised Manuscript with Track Changes'.

* An unmarked version of your revised paper without tracked changes. You should upload this as a separate file labeled 'Manuscript'.

If you would like to make changes to your financial disclosure, competing interests statement, or data availability statement, please make these updates within the submission form at the time of resubmission. Guidelines for resubmitting your figure files are available below the reviewer comments at the end of this letter

We look forward to receiving your revised manuscript.

Kind regards,

Fabian Spill

Academic Editor

PLOS Computational Biology

Mark Alber

Section Editor

PLOS Computational Biology

**Journal Requirements:**

1) We ask that a manuscript source file is provided at Revision. Please upload your manuscript file as a .doc, .docx, .rtf or .tex. If you are providing a .tex file, please upload it under the item type u2018LaTeX Source Fileu2019 and leave your .pdf version as the item type u2018Manuscriptu2019.

2) Please upload all main figures as separate Figure files in .tif or .eps format. For more information about how to convert and format your figure files please see our guidelines:

https://journals.plos.org/ploscompbiol/s/figures

3) We have noticed that you have uploaded Supporting Information files, but you have not included a list of legends. Please add a full list of legends for your Supporting Information files after the references list.

4) Please ensure that the funders and grant numbers match between the Financial Disclosure field and the Funding Information tab in your submission form. Note that the funders must be provided in the same order in both places as well.

- State the initials, alongside each funding source, of each author to receive each grant. For example: "This work was supported by the National Institutes of Health (####### to AM; ###### to CJ) and the National Science Foundation (###### to AM)."

- State what role the funders took in the study. If the funders had no role in your study, please state: "The funders had no role in study design, data collection and analysis, decision to publish, or preparation of the manuscript.".

If you did not receive any funding for this study, please simply state: u201cThe authors received no specific funding for this work.u201d

**Reviewers' comments:**

Reviewer's Responses to Questions

**Comments to the Authors:**

**Please note here if the review is uploaded as an attachment.**

Reviewer #1: ## Summary

Huber and Finley present a Bayesian framework for predicting kinetic parameters in mechanistic ODE models, with applications to ERBB and GPCR signaling pathways. While the topic is of clear interest and relevance, the study falls short in several key areas. The results lack sufficient strength to convincingly support the proposed approach. Moreover, the manuscript is difficult to follow due to its diffuse and at times disorganised presentation. A substantial rewrite is necessary to improve readability.

## Major Comments

### Technical Details & Presentation

- **Predictions**: The rationale for choosing SHC in the EGFR model is unconvincing. Given that many model species already have direct clinical relevance, predictions should be done in a leave-one-out fashion. This would also increase the number examples in the test set to something more meaningful.

- **Baselines**: The evaluation of predictions does not include any positive/negative baselines. How difficult/out-of-distribution are those predictions?

### **Overall Structure and Clarity**

- **Introduction Placement and Focus**: The comparison of this study with existing literature is more appropriate for the _Discussion_ section, where it can be contextualised against the findings. In its current position in the _Introduction_, it disrupts the logical flow.

- **Narrative Coherence**: The introductions frequently shifts between background information and descriptions of the current study, which affects readability.

- **Conciseness**: Explanations are often overly elaborate and could be significantly streamlined. I would expect that the text can be shortened by at least 25-50%.

### **Content and Terminology**

- **Dataset and Model Overview**: The manuscript would benefit from a clearly organised section that outlines the datasets used, the machine learning predictors employed, and the structure of the ODE models. This should be presented early in the methods or a dedicated overview section.

- **Terminology Use**: Avoid introducing technical terms such as _ybase_ and _yaug_ in the introduction. These are best introduced in the methods or results sections, where they can be explained in the context of their use.

- **Figure Descriptions**: Explanations related to figure layout and visual elements should be moved to the figure captions. The main text should focus on interpreting the results, not describing the figure composition.

- **Sections 2.5–2.7**: These sections do not add substantive value to the manuscript and could be removed or significantly condensed to maintain focus and improve pacing.

Reviewer #2: Please see attached file.

Reviewer #3: This work proposes to use available databases to improve parameter inference of models of cell signaling. In particular, they aim to augment sparse time-series data of concentrations (typically used for inference) with measurements of protein and amino acid sequence structure that have become available. This data is used in a ML pipeline that predicts binding affinity parameters of cell signaling models. Using a Bayesian inference approach and the KL divergence metric between distributions, they quantify the information gained from incorporating these additional sequence datasets. They apply their approach to EGFR and GPCR signaling models. The use of Alpha Fold and other ML models to bridge the scale of sequence/structure to the scale of protein concentrations was interesting and new to me. There is also the potential to generalize the approach to other models. Overall, the paper is well-written so I recommend minor revisions (that mostly have to do with incorporating background and methods information better into results) as follows:

1. On page 10, where the authors mention that “One of these _D predictions was made with an experimental structure”, it would be good to already clarify that the experimental structure was available for GPCR signaling, and that all others are based on AlphaFold predicted structures.

2. I think the manuscript would be improved if the authors mentioned earlier that their choice of the two test cases shows scenarios that vary in model complexity, in the number of unknown parameters, in the availability of experimental data, etc.

3. Section 2.3 begins quite abruptly with results of Bayesian inference, but not much is known at this point about the nature of the ODE models, the type of data used for parameter estimation, the data used for testing, etc. This is all addressed in Materials and Methods, but it feels like some of this background is needed here to properly understand the setting of the pipeline and results.

4. In figure 4c), is there any reason why the baseline distribution for non-binding parameters is tighter than for the other parameter categories?

5. In the paragraph starting on line 251, the authors discuss that output other than the “test set” may look qualitatively different in predictions from their baseline vs augmented parameter estimation. As mentioned earlier, it is not clear at this point what this means. While this is clarified later, I think it is important to mention it here to be able to distinguish between the different data predictions.

6. Figure 6 lists panel d) before panel c).

7. Typo on line 433, I think it should say “are more stringent than the default…”

8. In Materials and Methods, the authors say that “To calculate the projected change in an output due to a 5% change in a particular parameter, we multiple the local sensitivity by 5% of the maximum likelihood parameters.” I haven’t seen this approached in this way before. Is there a rigorous basis for doing this?

Reviewer #4: Summary: This study introduces a multiscale probabilistic framework that integrates machine learning-derived binding affinity estimates into mechanistic models of cell signaling. The authors use protein structure data from PDB and AlphaFold, along with sequence information from UniProt, to infer kinetic parameters, such as binding and unbinding rates, for receptor systems including EGFR and GPCRs. A data augmentation strategy incorporates predictions from protein structure models into the Bayesian inference process. Changes in the posterior distributions of binding parameters, including shifts in uncertainty and central estimates, are quantified using Kullback-Leibler divergence. These inferred parameters are then used to simulate downstream signaling dynamics, which are compared to experimental measurements to assess predictive consistency.

Strengths and innovation

1. Multiscale data integration: The framework bridges structural and systems biology by combining molecular-scale information, e.g., protein sequences and AlphaFold-predicted structures, with dynamic models of cell signaling.

2. Structure-informed parameter inference: The study incorporates binding affinity estimates derived from protein structure models, both experimental and predicted, into the inference of kinetic parameters, enabling application even in the absence of crystal structures.

3. Bayesian framework with Kullback-Leibler (KL)-based evaluation: A Bayesian approach is used to infer binding and unbinding rates, and changes in posterior distributions due to data augmentation are quantified using KL divergence.

4. Application to receptor signaling pathways: The method is demonstrated on EGFR and GPCR systems, showing a pipeline for integrating structural predictions into dynamic pathway modeling.

Areas of improvement

The approach proposed by the authors is highly creative: it leverages crystal structures, or AlphaFold-predicted structures when experimental data is unavailable, to estimate binding affinities, which are then used to inform parameter inference in signaling models. While this multiscale strategy is compelling, it also presents important challenges.

Inferring binding affinities from AlphaFold-derived structures is very challenging. AlphaFold and related structural models are designed to predict a single, static apo conformation, which may not correspond to the binding-competent state required for accurate kinetic parameter inference. This limitation is particularly pronounced for flexible proteins such as EGFR and GPCRs, where conformational heterogeneity is known to be essential for binding. Using only the lowest-energy apo structure may lead to inaccurate or biased estimates. To evaluate the robustness of their approach, the authors could apply their pipeline to both apo and holo structures from the PDB for EGFR and representative GPCRs. Comparing predicted KD values across conformational states would help determine the sensitivity of their predictions to structural flexibility and state-specific effects.

An even better test would involve identifying or generating molecular dynamics (MD) simulations of EGFR and representative GPCRs. Such simulations provide ensembles of conformations sampled across biologically relevant timescales, capturing the dynamic nature of these flexible proteins. The authors could apply their KD prediction pipeline to different conformations from the MD ensembles and quantify the variance in predicted KD values, hence, assessing whether the model meaningfully captures the impact of conformational heterogeneity on binding affinity, or whether relying on static structures systematically biases the predictions.

Figure 3: The figure suggests that the structure-informed ML pipeline improves prediction accuracy of binding affinity (KD) relative to a log-uniform prior. However, the sample size is small (n = 10 binding reactions), which constrains the statistical power of the Mann-Whitney U test (was the p-value computed using an exact test or an asymptotic approximation?). Second, the assumption of independence may not hold, particularly in the ML pipeline group, where multiple predictions per reaction (e.g., from different AlphaFold-generated structures) may have been included. Treating these as independent observations could artificially inflate statistical significance. Additionally, the high variance in prediction error, for both the uninformed baseline and the ML pipeline, raises concerns about the practical utility of the results. For instance, although the mean error for the ML pipeline is lower, the large standard deviation suggests that worse predictions remain reasonably likely. Overall, while the figure suggests improvement, stronger statistical validation and clearer definitions of replicates are needed.

Figure 4 evaluates the impact of data augmentation on parameter inference. Panel (a) shows 90% quantiles of marginal posterior distributions of binding parameters, indicating that augmented data generally narrows posterior distributions. While this suggests increased certainty, the direction of shift is not uniformly beneficial: for some parameters (e.g., k12b, k1f, k1b), the augmented posterior appears to move away from the ground truth. Could the authors apply a statistical test, such as a paired comparison of MAE across conditions or Bayesian model selection, to assess whether the augmented posteriors yield more accurate estimates, not just tighter ones? Panel (b) quantifies information gain using KL divergence, which reflects changes in distribution but not accuracy. While it confirms that data augmentation alters the posteriors compared to the baseline, it does not indicate whether the augmented estimates are closer to the true values. Panel (c) is more informative, showing that augmentation shifts the posterior distributions of unbinding parameters and reduces the mean absolute error (MAE), while binding and non-binding parameters remain largely unchanged. The lack of effect on non-binding parameters is expected, as the augmented data specifically targets binding interactions. However, the limited impact on binding parameters is less intuitive, given their coupling with unbinding rates. Could the authors clarify why augmentation selectively improves unbinding estimates? Is this due to the structure of the likelihood function, the nature of the ML predictions (e.g., affinity being more sensitive to unbinding), or another modeling constraint?

Figure 5 shows that data augmentation has only a moderate effect on the baseline model’s predictive performance. In the EGFR panel, neither model captures the experimental data well, particularly SHC phosphorylation dynamics, raising concerns about the adequacy of the model or its parameterization. The authors should clarify whether this mismatch stems from model limitations, training data quality, or the scope of the augmented information. In the GPCR panel, both models better match experimental data, but the differences are minimal (or at least, not observable in the figure). It remains unclear whether augmentation improves model fit. A quantitative comparison, e.g., RMSE, likelihood scores, or posterior predictive checks, would help determine whether the improvement is statistically significant.

Small comments:

The authors should provide a clear mathematical description of the signaling network model used for parameter inference, including the system of differential equations, model parameters, and underlying kinetic assumptions. This would clarify how structural priors influence dynamic outputs. Additionally, more detail on the Bayesian framework and the SVM-based PPI-Affinity model would improve transparency, specifically, information on the feature space, kernel type, and training data, which are essential for assessing prediction reliability and reproducibility.

**********

**Have the authors made all data and (if applicable) computational code underlying the findings in their manuscript fully available?**

The PLOS Data policy requires authors to make all data and code underlying the findings described in their manuscript fully available without restriction, with rare exception (please refer to the Data Availability Statement in the manuscript PDF file). The data and code should be provided as part of the manuscript or its supporting information, or deposited to a public repository. For example, in addition to summary statistics, the data points behind means, medians and variance measures should be available. If there are restrictions on publicly sharing data or code —e.g. participant privacy or use of data from a third party—those must be specified.

Reviewer #1: Yes

Reviewer #2: Yes

Reviewer #3: Yes

Reviewer #4: Yes

**********

PLOS authors have the option to publish the peer review history of their article (what does this mean?). If published, this will include your full peer review and any attached files.

If you choose “no”, your identity will remain anonymous but your review may still be made public.

**Do you want your identity to be public for this peer review?**     For information about this choice, including consent withdrawal, please see our Privacy Policy.

Reviewer #1: No

Reviewer #2: No

Reviewer #3: No

Reviewer #4: **Yes:**     Maria Rodriguez Martinez

[NOTE: If reviewer comments were submitted as an attachment file, they will be attached to this email and accessible via the submission site. Please log into your account, locate the manuscript record, and check for the action link "View Attachments". If this link does not appear, there are no attachment files.]

**Figure resubmission:**

While revising your submission, we strongly recommend that you use PLOS’s NAAS tool (https://ngplosjournals.pagemajik.ai/artanalysis) to test your figure files. NAAS can convert your figure files to the TIFF file type and meet basic requirements (such as print size, resolution), or provide you with a report on issues that do not meet our requirements and that NAAS cannot fix.

After uploading your figures to PLOS’s NAAS tool - https://ngplosjournals.pagemajik.ai/artanalysis, NAAS will process the files provided and display the results in the "Uploaded Files" section of the page as the processing is complete. If the uploaded figures meet our requirements (or NAAS is able to fix the files to meet our requirements), the figure will be marked as "fixed" above. If NAAS is unable to fix the files, a red "failed" label will appear above. When NAAS has confirmed that the figure files meet our requirements, please download the file via the download option, and include these NAAS processed figure files when submitting your revised manuscript.

**Reproducibility:**

To enhance the reproducibility of your results, we recommend that authors of applicable studies deposit laboratory protocols in protocols.io, where a protocol can be assigned its own identifier (DOI) such that it can be cited independently in the future. Additionally, PLOS ONE offers an option to publish peer-reviewed clinical study protocols. Read more information on sharing protocols at https://plos.org/protocols?utm_medium=editorial-email&utm_source=authorletters&utm_campaign=protocols

Attachment Submitted filename: PLCB_Huber.pdf

# Author comment: Author response to Decision Letter 1

**DOI:** 10.1371/journal.pcbi.1014321.r002

10 Dec 2025

Attachment Submitted filename: Response to Reviewers_final.docx

# Peer review: Decision Letter 1

**DOI:** 10.1371/journal.pcbi.1014321.r003

**Authors:** Fabian Spill

11 Jan 2026

PCOMPBIOL-D-25-01377R1

Multiscale Probabilistic Modeling: A Bayesian Approach to Augment Mechanistic Models of Cell Signaling with Machine-Learning Predictions of Binding Affinity

PLOS Computational Biology

Dear Dr. Finley,

Thank you for submitting your manuscript to PLOS Computational Biology. After careful consideration, we feel that it has merit but does not fully meet PLOS Computational Biology's publication criteria as it currently stands. Therefore, we invite you to submit a revised version of the manuscript that addresses the points raised during the review process.

Please submit your revised manuscript by Mar 13 2026 11:59PM. If you will need more time than this to complete your revisions, please reply to this message or contact the journal office at ploscompbiol@plos.org. When you're ready to submit your revision, log on to https://www.editorialmanager.com/pcompbiol/ and select the 'Submissions Needing Revision' folder to locate your manuscript file.

Please include the following items when submitting your revised manuscript:

* A letter that responds to each point raised by the editor and reviewer(s). You should upload this letter as a separate file labeled 'Response to Reviewers'. This file does not need to include responses to formatting updates and technical items listed in the 'Journal Requirements' section below.

* A marked-up copy of your manuscript that highlights changes made to the original version. You should upload this as a separate file labeled 'Revised Manuscript with Track Changes'.

* An unmarked version of your revised paper without tracked changes. You should upload this as a separate file labeled 'Manuscript'.

If you would like to make changes to your financial disclosure, competing interests statement, or data availability statement, please make these updates within the submission form at the time of resubmission. Guidelines for resubmitting your figure files are available below the reviewer comments at the end of this letter.

We look forward to receiving your revised manuscript.

Kind regards,

Fabian Spill

Academic Editor

PLOS Computational Biology

Mark Alber

Section Editor

PLOS Computational Biology

**Additional Editor Comments:**

Reviewer 1 and 2 still have some important points which the authors should address, together with the minor comments, before the paper can be formally accepted

**Journal Requirements:**

If the reviewer comments include a recommendation to cite specific previously published works, please review and evaluate these publications to determine whether they are relevant and should be cited. There is no requirement to cite these works unless the editor has indicated otherwise.

**Reviewers' comments:**

Reviewer's Responses to Questions

**Comments to the Authors:**

**Please note here if the review is uploaded as an attachment.**

Reviewer #1: I thank the authors for substantially revising the manuscript; the text now reads much more smoothly and has markedly improved flow. While I find the overarching idea of the manuscript strong, the current results leave me with the impression that, at least in its present form, the combined approach does not yet demonstrate a clear improvement in predictive performance.

## Major concerns

### ML pipeline

It is important to clarify (and ideally explicitly verify) that the PPI affinity model was not trained on the same K_D parameters used in this study. Given the stated publication date (2022), it seems plausible that AlphaFold3-predicted structures were not available, but I cannot follow the authors’ claim that no PDB structures were available for any of the complexes in the EGFR model. A quick search yields several relevant structures, for example:

- EGF:EGFR – https://www.rcsb.org/structure/1IVO, https://www.rcsb.org/structure/8HGS

- SHC1:EGFR(L858R) – https://www.rcsb.org/structure/5CZI

- GRB2:SOS1 (peptides) – https://www.rcsb.org/structure/1GBR

Moreover, PPI affinity appears to be intended primarily for protein–peptide binding prediction, whereas the Materials and Methods suggest that full-length sequences of both interactors were used. Together with the fact that many of these interactions are weak, transient, and often mediated by intrinsically disordered regions (precisely the types of interactions where AlphaFold-derived approaches are known to struggle), this warrants a substantially more careful validation of the ML pipeline and its applicability to the present setting.

### Combined pipeline

From Figures 5 and 6, it appears that although parameter estimates improve, the model predictions themselves remain essentially unchanged. This is somewhat disappointing, as it would suggest that the combined mechanistic + ML approach yields limited practical gain beyond parameter regularisation.

One possibility is that the inferred K_D values are non-identifiable, and that the augmentation primarily constrains the ratio of binding and unbinding rates to match the predicted K_D, without altering predictive behavior. This could be tested directly by comparing the baseline, augmented, and ML predicted K_D distributions.

## Minor concerns

- Lines 254, 286, 289: “Error! Reference source not found.”

Reviewer #2: The authors have addressed the majority of my original concerns. The introduction, in particular, is now much more clear.

I am still unconvinced that the ML approach augments the likelihood, although I accept that it probably doesn't matter. Specifically, the output of the ML approach is a predicted combination of the model parameters. I would see this more as adjusting the prior, which is then used with an explicit likelihood to formulate the posterior.

Perhaps the authors could explicitly show how they (mathematically) augment the likelihood with the output of the ML pipeline (e..g., to backup lines 350-352 and Section 4.6). Aon line 521 the authors state that the full details are included in the supplementary information, however the text provided in the SI is not explicit (and again makes it look like a prior placed on K_D).

A final minor comment is that it is unclear to me what the authors mean on line 60 when they say that "data on protein sequence is on a different scale". Compared to what? Scale as in the number of data points/reads? Or the measurement scale of the quantities they are interested in?

Reviewer #4: The revision is substantially improved, and the authors’ responses to the reviewers are clear and generally satisfactory. I recommend acceptance.

As a minor, forward-looking note, I encourage the authors to briefly acknowledge in the Discussion the limitations of inferring

K_D from AlphaFold-derived structures, particularly in contexts where binding involves conformational selection and/or induced-fit mechanisms upon complex formation. However, I recognize that a deeper analysis may be beyond the scope of the current manuscript, and hence, my overall recommendation is acceptance.

**********

**Have the authors made all data and (if applicable) computational code underlying the findings in their manuscript fully available?**

The PLOS Data policy requires authors to make all data and code underlying the findings described in their manuscript fully available without restriction, with rare exception (please refer to the Data Availability Statement in the manuscript PDF file). The data and code should be provided as part of the manuscript or its supporting information, or deposited to a public repository. For example, in addition to summary statistics, the data points behind means, medians and variance measures should be available. If there are restrictions on publicly sharing data or code —e.g. participant privacy or use of data from a third party—those must be specified.

Reviewer #1: Yes

Reviewer #2: None

Reviewer #4: Yes

**********

PLOS authors have the option to publish the peer review history of their article (what does this mean?). If published, this will include your full peer review and any attached files.

If you choose “no”, your identity will remain anonymous but your review may still be made public.

**Do you want your identity to be public for this peer review?**     For information about this choice, including consent withdrawal, please see our Privacy Policy.

Reviewer #1: No

Reviewer #2: **Yes:**     Alexander Browning

Reviewer #4: **Yes:**     Maria Rodriguez Martinez

[NOTE: If reviewer comments were submitted as an attachment file, they will be attached to this email and accessible via the submission site. Please log into your account, locate the manuscript record, and check for the action link "View Attachments". If this link does not appear, there are no attachment files.]

**Figure resubmission:**

-->While revising your submission, we strongly recommend that you use PLOS’s NAAS tool (https://ngplosjournals.pagemajik.ai/artanalysis) to test your figure files. NAAS can convert your figure files to the TIFF file type and meet basic requirements (such as print size, resolution), or provide you with a report on issues that do not meet our requirements and that NAAS cannot fix.

After uploading your figures to PLOS’s NAAS tool - https://ngplosjournals.pagemajik.ai/artanalysis, NAAS will process the files provided and display the results in the "Uploaded Files" section of the page as the processing is complete. If the uploaded figures meet our requirements (or NAAS is able to fix the files to meet our requirements), the figure will be marked as "fixed" above. If NAAS is unable to fix the files, a red "failed" label will appear above. When NAAS has confirmed that the figure files meet our requirements, please download the file via the download option, and include these NAAS processed figure files when submitting your revised manuscript.

**Reproducibility:**

To enhance the reproducibility of your results, we recommend that authors of applicable studies deposit laboratory protocols in protocols.io, where a protocol can be assigned its own identifier (DOI) such that it can be cited independently in the future. Additionally, PLOS ONE offers an option to publish peer-reviewed clinical study protocols. Read more information on sharing protocols at https://plos.org/protocols?utm_medium=editorial-email&utm_source=authorletters&utm_campaign=protocols

# Author comment: Author response to Decision Letter 2

**DOI:** 10.1371/journal.pcbi.1014321.r004

1 Apr 2026

Attachment Submitted filename: Response to Reviewers_R2.docx

# Peer review: Decision Letter 2

**DOI:** 10.1371/journal.pcbi.1014321.r005

**Authors:** Fabian Spill

29 Apr 2026

PCOMPBIOL-D-25-01377R2

Multiscale Probabilistic Modeling: A Bayesian Approach to Augment Mechanistic Models of Cell Signaling with Machine-Learning Predictions of Binding Affinity

PLOS Computational Biology

Dear Dr. Finley,

Thank you for submitting your manuscript to PLOS Computational Biology. After careful consideration, we feel that it has merit but does not fully meet PLOS Computational Biology's publication criteria as it currently stands. Therefore, we invite you to submit a revised version of the manuscript that addresses the points raised during the review process.

Please submit your revised manuscript by Jun 29 2026 11:59PM. If you will need more time than this to complete your revisions, please reply to this message or contact the journal office at ploscompbiol@plos.org. When you're ready to submit your revision, log on to https://www.editorialmanager.com/pcompbiol/ and select the 'Submissions Needing Revision' folder to locate your manuscript file.

Please include the following items when submitting your revised manuscript:

* A letter that responds to each point raised by the editor and reviewer(s). You should upload this letter as a separate file labeled 'Response to Reviewers'. This file does not need to include responses to formatting updates and technical items listed in the 'Journal Requirements' section below.

* A marked-up copy of your manuscript that highlights changes made to the original version. You should upload this as a separate file labeled 'Revised Manuscript with Track Changes'.

* An unmarked version of your revised paper without tracked changes. You should upload this as a separate file labeled 'Manuscript'.

If you would like to make changes to your financial disclosure, competing interests statement, or data availability statement, please make these updates within the submission form at the time of resubmission. Guidelines for resubmitting your figure files are available below the reviewer comments at the end of this letter.

We look forward to receiving your revised manuscript.

Kind regards,

Fabian Spill

Academic Editor

PLOS Computational Biology

Mark Alber

Section Editor

PLOS Computational Biology

**Additional Editor Comments:**

One reviewier has a minor point of clarification. If the authors could kindly address this point, and then the manuscript can be formally accepted.

**Reviewers' comments:**

Reviewer's Responses to Questions

**Comments to the Authors:**

**Please note here if the review is uploaded as an attachment.**

Reviewer #1: The authors have reasonably addressed most of my concerns.

Reviewer #2: I thank the authors for addressing the majority of my remaining comments. I only have one minor point below that could be clarified before publication.

I can see now that the authors construct a likelihood for K_D using a log normal distribution along with the ML predicted estimate (through f(theta)) and the ML predicted error (sigma). It would be helpful if the authors could be a little more explicit about their assumptions and the transformation from an error estimate to the standard deviation of exp(K_D). Are the estimates (error and point) from the ML approach on the log scale already? Or do the authors argue through the order-of-magnitude change that they are already on the log scale? Particular as, at first glance, the mean and standard deviation are not given by f(theta) and sigma, respectively, but rather transformations thereof.

**********

**Have the authors made all data and (if applicable) computational code underlying the findings in their manuscript fully available?**

The PLOS Data policy requires authors to make all data and code underlying the findings described in their manuscript fully available without restriction, with rare exception (please refer to the Data Availability Statement in the manuscript PDF file). The data and code should be provided as part of the manuscript or its supporting information, or deposited to a public repository. For example, in addition to summary statistics, the data points behind means, medians and variance measures should be available. If there are restrictions on publicly sharing data or code —e.g. participant privacy or use of data from a third party—those must be specified.

Reviewer #1: Yes

Reviewer #2: Yes

**********

PLOS authors have the option to publish the peer review history of their article (what does this mean?). If published, this will include your full peer review and any attached files.

If you choose “no”, your identity will remain anonymous but your review may still be made public.

**Do you want your identity to be public for this peer review?**     For information about this choice, including consent withdrawal, please see our Privacy Policy.

Reviewer #1: No

Reviewer #2: No

[NOTE: If reviewer comments were submitted as an attachment file, they will be attached to this email and accessible via the submission site. Please log into your account, locate the manuscript record, and check for the action link "View Attachments". If this link does not appear, there are no attachment files.]

**Figure resubmission:**

-->While revising your submission, we strongly recommend that you use PLOS’s NAAS tool (https://ngplosjournals.pagemajik.ai/artanalysis) to test your figure files. NAAS can convert your figure files to the TIFF file type and meet basic requirements (such as print size, resolution), or provide you with a report on issues that do not meet our requirements and that NAAS cannot fix.

After uploading your figures to PLOS’s NAAS tool - https://ngplosjournals.pagemajik.ai/artanalysis, NAAS will process the files provided and display the results in the "Uploaded Files" section of the page as the processing is complete. If the uploaded figures meet our requirements (or NAAS is able to fix the files to meet our requirements), the figure will be marked as "fixed" above. If NAAS is unable to fix the files, a red "failed" label will appear above. When NAAS has confirmed that the figure files meet our requirements, please download the file via the download option, and include these NAAS processed figure files when submitting your revised manuscript.

**Reproducibility:**

To enhance the reproducibility of your results, we recommend that authors of applicable studies deposit laboratory protocols in protocols.io, where a protocol can be assigned its own identifier (DOI) such that it can be cited independently in the future. Additionally, PLOS ONE offers an option to publish peer-reviewed clinical study protocols. Read more information on sharing protocols at https://plos.org/protocols?utm_medium=editorial-email&utm_source=authorletters&utm_campaign=protocols

# Author comment: Author response to Decision Letter 3

**DOI:** 10.1371/journal.pcbi.1014321.r006

8 May 2026

Attachment Submitted filename: Response to Reviewers_R3.docx

# Editor report: Decision Letter 3

**DOI:** 10.1371/journal.pcbi.1014321.r007

**Authors:** Fabian Spill

11 May 2026

Dear Professor Finley,

We are pleased to inform you that your manuscript 'Multiscale Probabilistic Modeling: A Bayesian Approach to Augment Mechanistic Models of Cell Signaling with Machine-Learning Predictions of Binding Affinity' has been provisionally accepted for publication in PLOS Computational Biology.

Before your manuscript can be formally accepted you will need to complete some formatting changes, which you will receive in a follow up email. A member of our team will be in touch with a set of requests.

Please note that your manuscript will not be scheduled for publication until you have made the required changes, so a swift response is appreciated.

IMPORTANT: The editorial review process is now complete. PLOS will only permit corrections to spelling, formatting or significant scientific errors from this point onwards. Requests for major changes, or any which affect the scientific understanding of your work, will cause delays to the publication date of your manuscript.

Should you, your institution's press office or the journal office choose to press release your paper, you will automatically be opted out of early publication. We ask that you notify us now if you or your institution is planning to press release the article. All press must be co-ordinated with PLOS.

Thank you again for supporting Open Access publishing; we are looking forward to publishing your work in PLOS Computational Biology.

Best regards,

Fabian Spill

Academic Editor

PLOS Computational Biology

Mark Alber

Section Editor

PLOS Computational Biology

***********************************************************

# Editor report: Acceptance letter

**DOI:** 10.1371/journal.pcbi.1014321.r008

**Authors:** Fabian Spill

PCOMPBIOL-D-25-01377R3

A Multiscale, Bayesian Inference Approach to Augment Mechanistic Models of Cell Signaling with Machine-Learning Predictions of Binding Affinity

Dear Dr Finley,

I am pleased to inform you that your manuscript has been formally accepted for publication in PLOS Computational Biology. Your manuscript is now with our production department and you will be notified of the publication date in due course.

The corresponding author will soon be receiving a typeset proof for review, to ensure errors have not been introduced during production. Please review the PDF proof of your manuscript carefully, as this is the last chance to correct any errors. Please note that major changes, or those which affect the scientific understanding of the work, will likely cause delays to the publication date of your manuscript.

Soon after your final files are uploaded, unless you have opted out, the early version of your manuscript will be published online. The date of the early version will be your article's publication date. The final article will be published to the same URL, and all versions of the paper will be accessible to readers.

For Research, Software, and Methods articles, you will receive an invoice from PLOS for your publication fee after your manuscript has reached the completed accept phase. If you receive an email requesting payment before acceptance or for any other service, this may be a phishing scheme. Learn how to identify phishing emails and protect your accounts at https://explore.plos.org/phishing.

Thank you again for supporting PLOS Computational Biology and open-access publishing. We are looking forward to publishing your work!

With kind regards,

Anita Estes

PLOS Computational Biology | Carlyle House, Carlyle Road, Cambridge CB4 3DN | United Kingdom ploscompbiol@plos.org | Phone +44 (0) 1223-442824 | ploscompbiol.org | @PLOSCompBiol
